# Reprogramming dysfunctional CD8^+^ T cells to promote properties associated with natural HIV control

**DOI:** 10.1172/JCI157549

**Published:** 2022-06-01

**Authors:** Federico Perdomo-Celis, Caroline Passaes, Valérie Monceaux, Stevenn Volant, Faroudy Boufassa, Pierre de Truchis, Morgane Marcou, Katia Bourdic, Laurence Weiss, Corinne Jung, Christine Bourgeois, Cécile Goujard, Laurence Meyer, Michaela Müller-Trutwin, Olivier Lambotte, Asier Sáez-Cirión

**Affiliations:** 1 Institut Pasteur, Université Paris Cité, Unité HIV Inflammation et Persistance, Paris, France.; 2 Institut Pasteur, Université Paris Cité, Hub Bioinformatique et Biostatistique, Paris, France.; 3 Université Paris Saclay, INSERM Centre de Recherche en Épidémiologie et Santé des Populations (CESP) U1018, Assistance Publique–Hôpitaux de Paris (AP-HP), Department of Public Health, Bicêtre Hospital, Paris, France.; 4 Université Paris-Saclay, AP-HP Hôpital Raymond Poincaré, Garches, France.; 5 Université Paris-Saclay, AP-HP, Bicêtre Hospital, UMR1184 INSERM Commissariat à l’énergie atomique et aux énergies alternatives (CEA), Le Kremlin Bicêtre, France.; 6 Université de Paris Cité, AP-HP, Paris Centre, Hôtel Dieu, Paris, France.; 7 Université Paris-Saclay, AP-HP, Hôpital Bicêtre, Départements Médico-Universitaires (DMU) 7, INSERM U1018, CESP, Le Kremlin Bicêtre, France.

**Keywords:** AIDS/HIV, Immunology, Adaptive immunity, Cellular immune response, Immunotherapy

## Abstract

Virus-specific CD8^+^ T cells play a central role in HIV-1 natural controllers to maintain suppressed viremia in the absence of antiretroviral therapy. These cells display a memory program that confers them stemness properties, high survival, polyfunctionality, proliferative capacity, metabolic plasticity, and antiviral potential. The development and maintenance of such qualities by memory CD8^+^ T cells appear crucial to achieving natural HIV-1 control. Here, we show that targeting the signaling pathways Wnt/transcription factor T cell factor 1 (Wnt/TCF-1) and mTORC through GSK3 inhibition to reprogram HIV-specific CD8^+^ T cells from noncontrollers promoted functional capacities associated with natural control of infection. Features of such reprogrammed cells included enrichment in TCF-1^+^ less-differentiated subsets, a superior response to antigen, enhanced survival, polyfunctionality, metabolic plasticity, less mTORC1 dependency, an improved response to γ-chain cytokines, and a stronger HIV-suppressive capacity. Thus, such CD8^+^ T cell reprogramming, combined with other available immunomodulators, might represent a promising strategy for adoptive cell therapy in the search for an HIV-1 cure.

## Graphical abstract

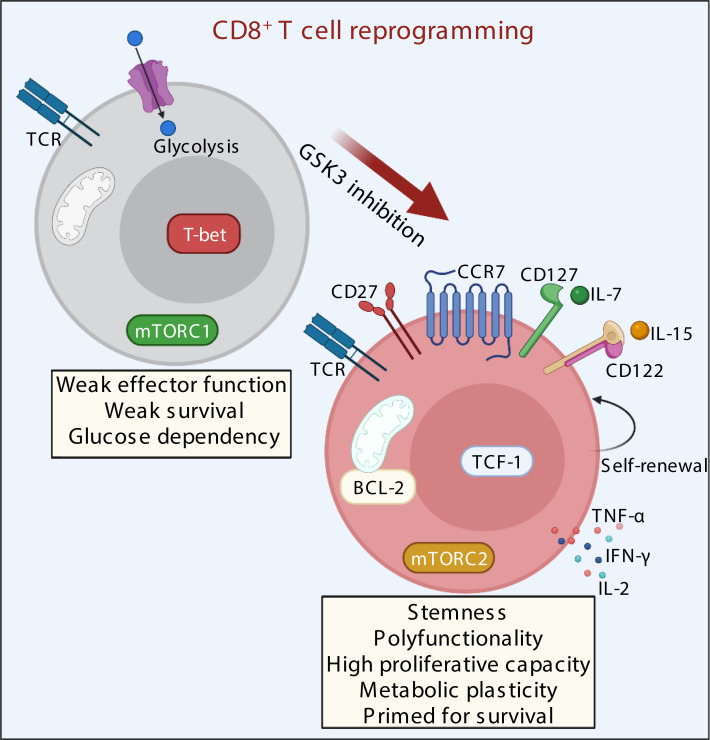

## Introduction

For activation and differentiation, CD8^+^ T cells require T cell receptor (TCR) signals provided by peptide/MHC class I, together with costimulation, and cytokines. The amount and duration of these signals influence the priming and fate of memory CD8^+^ T cells (1). HIV-1 infection is an example of the different outcomes a CD8^+^ T cell can reach according to the quality of the priming received early after infection. For instance, HIV-1 controllers (HICs), who represent less than 0.5% of people living with HIV-1, have controlled viremia for long periods in the absence of antiretroviral therapy (ART), and such control is associated with strong HIV-specific CD8^+^ T cell responses (2, 3). Compelling studies indicate that HIV-specific CD8^+^ T cells from HICs exhibit stemness properties, high survival, proliferative potential, and polyfunctionality upon antigen stimulation (4–7). Recently, we demonstrated that HIV-specific central memory CD8^+^ T cells from HICs have a stem-like transcriptional program characterized by enhanced polyfunctionality and survival compared with cells from noncontrollers on ART (8). Moreover, we reported that early in primary SIV infection, there is a divergence in the memory compartment of SIV-specific CD8^+^ T cells of macaques that later in the chronic phase had naturally controlled or no control of infection. In this animal model, SIV controllers developed stem-like, SIV-specific CD8^+^ T cells, which preceded the optimal maturation of the CD8^+^ T cell response (i.e., acquisition of SIV-suppressive properties) during infection (9). Contrary to cells from HICs or SIV controllers, virus-specific memory CD8^+^ T cells from noncontrollers exhibit an effector-like and exhausted profile, limited survival capacity, and poor antiviral potential throughout infection (8–10). Overall, these observations are consistent with tuned priming of virus-specific CD8^+^ T cells early after infection in natural controllers, promoting long-lived, stem-like memory responses that mature throughout infection, whereas a skewed or nonregulated CD8^+^ T cell priming occurs in noncontrollers that promotes short-lived effector-like responses.

In part, the stem-like profile of memory CD8^+^ T cells is mediated by the transcription factor TCF-1, a downstream component of the Wnt/β-catenin pathway (11). As such, TCF-1 regulates stemness properties of CD8^+^ T cells, including longevity, self-renewal, and the potential to differentiate into multiple subsets (12). Evidence from chronic infection models indicates that stem-like memory TCF-1^+^CD8^+^ T cells have an enhanced capacity to contain infection and respond to secondary viral challenges (13–15). Fittingly, we and others have demonstrated that virus-specific CD8^+^ T cells from HIV-1 and SIV controllers have higher levels of TCF-1 compared with cells from noncontrollers (7, 9, 16), further suggesting that stem-like memory cells play a protective role in HIV-1 and SIV infections.

Several metabolic cues, such as activation of the mammalian target of rapamycin complex 1 and 2 (mTORC1/-2), also regulate CD8^+^ T cell fate, longevity, and effector functions (17). Constitutive activation of the mTORC1 pathway results in terminal differentiation of CD8^+^ T cells and a lack of long-term memory, whereas inhibition of mTORC2 leads to excessive activation and cell death (18). Accordingly, mTORC1 inhibition with rapamycin increases the number, quality, and survival of stem-like memory CD8^+^ T cells (19, 20). In line with those data, we previously showed that HIV-specific memory CD8^+^ T cells from HICs maintain metabolic plasticity and preferential activation of the mTORC2 pathway, whereas cells from noncontrollers are more dependent on glycolysis and mTORC1 (8). Thus, the ability to use diverse metabolic resources, as well as the suitable engagement of mTORC1 and 2 pathways, contributes to the enhanced functionality and longevity of memory HIV-specific CD8^+^ T cells from HICs.

Having identified some signaling pathways and metabolic cues that appear important for natural control of HIV-1 and SIV, we hypothesized that reprogramming of HIV-specific CD8^+^ T cells from noncontrollers to exhibit characteristics of CD8^+^ T cells of natural controllers could boost their antiviral potential. We recently showed that metabolic reprogramming with IL-15 increases mitochondrial respiration in CD8^+^ T cells from noncontrollers, contributing to improvement of the virus-suppressive capacity of these cells ex vivo (8). In the present study, we evaluated the potential of CD8^+^ T cell reprogramming to induce stem-like properties in cells derived from noncontroller individuals, via manipulation of the TCF-1 and mTORC pathways. We found that, after reprogramming, virus-specific CD8^+^ T cells from noncontrollers acquired phenotypic, transcriptional, metabolic, and functional attributes associated with natural control of HIV-1 infection. Our results shed light on the therapeutic potential of such CD8^+^ T cell reprogramming in the search for an HIV-1 cure.

## Results

### CD8^+^ T cell reprogramming toward a stem-like memory profile.

**Figure 1:**
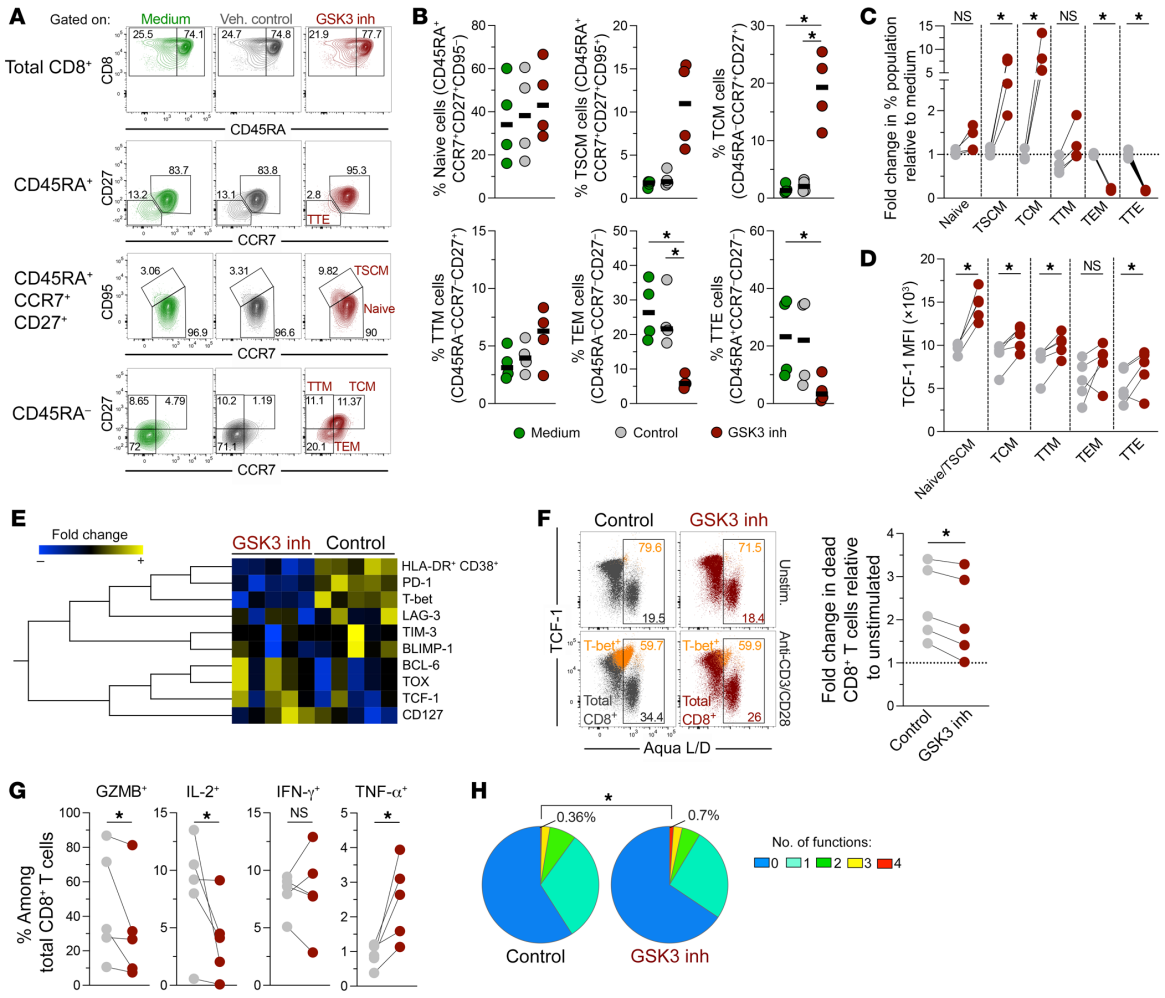
Induction of stem-like CD8^+^ T cells with high survival capacity and polyfunctionality by in vitro reprogramming. Total CD8^+^ T cells from individuals without HIV were treated with medium, vehicle (Veh.) control, or the GSK3 inhibitor (inh), followed by incubation under basal conditions or with anti-CD3/anti-CD28 stimulation for 48 hours. (**A** and **B**) Analysis of CD8^+^ T cell subpopulations in unstimulated cells (*n =* 4). (**C**) Fold change of CD8^+^ T cell subpopulations upon vehicle control or GSK3 inhibitor treatment, relative to the medium alone condition (*n =* 4). (**D**) Expression of TCF-1 in CD8^+^ T cell subsets (*n =* 4). (**E**) Fold change in the expression of the indicated markers induced by anti-CD3/anti-CD28 antibody stimulation relative to unstimulated cells (*n =* 5). (**F**) Analysis of dead cells by Aqua LIVE/DEAD^+^ staining (Aqua L/D) among total and T-bet^+^CD8^+^ T cells, and fold change in dead CD8^+^ T cells induced by anti-CD3/anti-CD28 stimulation relative to the unstimulated (Unstim.) condition (*n =* 5). (**G**) Frequencies of granzyme B^+^ (GZMB^+^), IL-2^+^, IFN-γ^+^, and TNF-α^+^ CD8^+^ T cells after anti-CD3/anti-CD28 stimulation. (**H**) Expression of 1 to 4 functions in CD8^+^ T cells (*n =* 5). *P < 0.05, by Dunn’s test (**B**) and Wilcoxon test (**C**, **D**, and **F**–**H**). Data obtained from 2 (**A**–**F**) or 3 (**G** and **H**) independent experiments are shown.

To test the hypothesis that CD8^+^ T cell reprogramming can invigorate stem-like properties, we used the glycogen synthase kinase 3 (GSK3) inhibitor 6-bromoindirubin-3′-oxime (BIO), which modulates pathways involved in the generation and maintenance of stem-like CD8^+^ T cells, such as the Wnt/β-catenin and mTORC2 pathways (12), that we and others have found to be upregulated in CD8^+^ T cells from natural controllers (7, 8, 16). We first characterized the effect of BIO-mediated reprogramming on bulk CD8^+^ T cells from individuals without HIV in terms of the memory phenotype (Supplemental Figure 1A; supplemental material available online with this article; https://doi.org/10.1172/JCI157549DS1). Of note, we performed dose-effect experiments to determine the optimal dose and duration of BIO treatment. We chose 3 μM and a 12-hour incubation, because under these conditions, we observed the greatest effects on the expression of representative phenotypic markers and no drug-induced cellular toxicity (Supplemental Figure 1B). Treatment with BIO (in the absence of further stimulation) was accompanied by the upregulation of surface expression of CCR7 and CD27 (Figure 1A). Accordingly, we observed an enrichment of CD8^+^ T cells with a less-differentiated stem cell memory (TSCM) and central memory (TCM) phenotype and a decrease in more-differentiated effector memory (TEM) and terminal effector (TTE) cells (Figure 1, A–C). We confirmed the effect of the GSK3 inhibitor by the enhanced expression of TCF-1 (Figure 1D). To confirm that these changes were related to the regulation of GSK3 activity, we also assessed the impact of TWS119, another GSK3 inhibitor previously used to promote stem-like memory CD8^+^ T cells (21). The treatment with both GSK3 inhibitors under parallel experimental conditions induced the expression of CCR7, CD27, and TCF-1 (Supplemental Figure 1C), increased TSCM and TCM cell populations, and decreased TEM cell populations (Supplemental Figure 1D). However, BIO consistently had a greater effect than did TWS119 (Supplemental Figure 1, C and D) and was used throughout the study. These results confirm that transient exposure to a GSK3 inhibitor, hereinafter referred to as CD8^+^ T cell reprogramming, promotes a memory-like phenotype in CD8^+^ T cells.

We wondered whether reprogrammed CD8^+^ T cells maintained their memory-like profile upon activation. Following anti-CD3/anti-CD28 stimulation, reprogrammed CD8^+^ T cells, when compared with cells in the control condition, exhibited lower expression of the activation markers HLA-DR and CD38; the inhibitory receptors programmed cell death 1 (PD-1), lymphocyte-activating 3 (LAG-3), and T cell immunoglobulin and mucin domain–containing protein 3 (TIM-3); and the transcription factors T-bet and B lymphocyte–induced maturation protein 1 (BLIMP-1), which are associated with an effector CD8^+^ T cell profile (Figure 1E, Supplemental Figure 1E, and ref. 22). On the contrary, reprogrammed CD8^+^ T cells maintained higher levels of the transcription factors BCL-6 and TCF-1, which are associated with a memory profile (23, 24), as well as CD127, which is critical for CD8^+^ T cell survival (Figure 1E and ref. 25). In addition, reprogrammed cells exhibited higher expression of thymocyte selection-associated high mobility group box (TOX). While this transcription factor regulates the transcriptional and epigenetic signature of exhausted T cells (26), it also restricts terminal effector differentiation and allows the persistence of antiviral CD8^+^ T cells (27, 28), and is found in polyfunctional memory CD8^+^ T cells (16). Consistent with the higher expression of CD127 and restriction of T-bet, reprogrammed CD8^+^ T cells showed lower levels of activation-induced cell death than did cells under the control condition (Figure 1F). We also observed the effects of CD8^+^ T cell reprogramming in the setting of either lower or higher TCR-mediated stimulation with anti-CD3/anti-CD28, with or without ICAM-1 (29), since reprogrammed CD8^+^ T cells maintained lower frequencies of T-bet^+^ cells than did nonreprogrammed cells (Supplemental Figure 1, F and G).

### Reprogrammed CD8^+^ T cells show enhanced polyfunctionality.

Since reprogramming of CD8^+^ T cells decreased the proportion of effector cells and restricted cell activation, we wondered whether reprogrammed CD8^+^ T cells could acquire functional capacity. Consistent with the restriction of an effector-like transcriptional program, reprogrammed total CD8^+^ T cells had lower expression of granzyme B and IL-2 than did nonreprogrammed cells upon stimulation with anti-CD3/anti-CD28, although we detected no change in IFN-γ production (Figure 1G). In contrast, there was a marked increase in the capacity of reprogrammed CD8^+^ T cells to produce TNF-α and overall increased polyfunctionality (Figure 1, G and H). We did not observe significant differences between nonreprogrammed and reprogrammed cells in the expression of the effector molecules on cells responding to simulation on a per-cell basis (e.g., fluorescence intensity of granzyme B in granzyme B^+^ cells) (Supplemental Figure 1H). Higher polyfunctionality was also observed after stimulation with anti-CD3/anti-CD28 plus ICAM-1 (Supplemental Figure 1I).

We also assessed the effect of CD8^+^ T cell reprogramming on proliferative capacity and effector maturation upon sequential polyclonal restimulation. Interestingly, reprogramming did not affect the capacity of CD8^+^ T cells to execute 1 to 3 division cycles, but prevented extensive proliferation (≥4 cell divisions) and maintained a higher proportion of quiescent cells (Supplemental Figure 1J). This pattern of regulated proliferation of reprogrammed cells was associated with lower coexpression of PD-1, LAG-3, TIM-3, and T cell immunoreceptor with Ig and ITIM domains (TIGIT) on CD8^+^ T cells after restimulation (Supplemental Figure 1K). Notably, we found that reprogramming did not irreversibly arrest effector maturation of less-differentiated TCM and transitional memory (TTM) cells, since these subsets could readily upregulate T-bet in the restimulation setting (Supplemental Figure 1L). Collectively, these results indicate that CD8^+^ T cell reprogramming induced TNF-α production and enhanced polyfunctionality, while maintaining responsiveness to recall.

### Reprogramming modifies the functional properties and transcriptional signature of all CD8^+^ T cell memory subsets.

**Figure 2:**
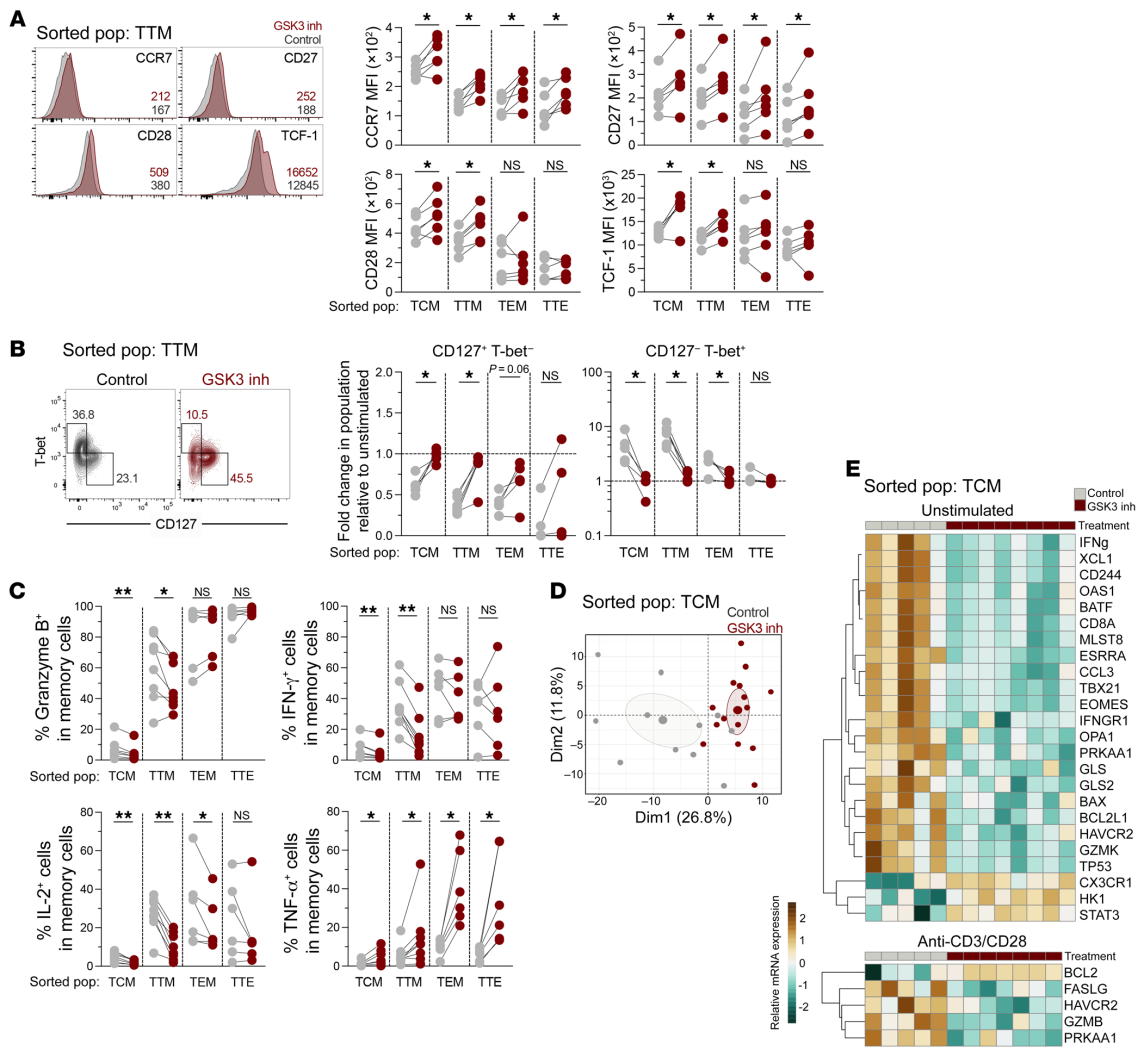
Intrinsic effects of reprogramming in CD8^+^ T cell memory subpopulations. Sorted TCM, TTM, TEM, and TTE cells from people without HIV were treated with the GSK3 inhibitor or vehicle control, followed by incubation under basal conditions or anti-CD3/anti-CD28 stimulation for 48 hours. (**A**) Representative histograms showing the expression of CCR7, CD27, CD28, and TCF-1 in sorted TTM cell populations (pop) under basal conditions. The median fluorescence intensity of each marker is indicated. Analysis of the expression of each marker in memory T cells after vehicle control or GSK3 inhibitor treatment, in the absence of stimulation, is shown. *P < 0.05, by Wilcoxon test. (**B**) Flow cytometric analysis of CD127^+^T-bet^–^ and CD127^–^T-bet^+^ cells after anti-CD3/anti-CD28 stimulation, and fold change in the indicated subsets among memory T cells induced by anti-CD3/anti-CD28 stimulation, relative to the unstimulated condition. *P < 0.05, by Wilcoxon test. (**C**) Frequencies of granzyme B^+^, IFN-γ^+^, IL-2^+^, and TNF-α^+^ cells among memory cells after anti-CD3/anti-CD28 stimulation. At least 5 donors were included for each comparison; data shown are from 7 independent experiments. *P < 0.05 and **P < 0.01, by Wilcoxon test. (**D**) PCA of gene expression by TCM cells treated with vehicle control or the GSK3 inhibitor. (**E**) Heatmap of genes differentially expressed (*P <* 0.05 according to a mixed-effects model) in reprogrammed versus nonreprogrammed TCM cells. Heatmap scale indicates mRNA levels relative to the housekeeping gene *GAPDH* and are plotted as log 2^–ΔCt^ in unstimulated and polyclonally stimulated cells (at least 5 donors were included for each comparison; data are from 2 independent experiments).

We asked whether the changes induced by cell reprogramming were related to phenotype conversion between CD8^+^ T cell subpopulations or if they were intrinsic to each subset. To answer this question, we isolated TCM, TTM, TEM, and TTE CD8^+^ T cells and treated them with the GSK3 inhibitor, followed by resting or stimulation with anti-CD3/anti-CD28 antibodies. In basal conditions, we found that cell reprogramming caused the upregulation of CCR7 and CD27 in all cell subsets (Figure 2A). Thus, the enrichment in less-differentiated cells observed after CD8^+^ T cell reprogramming (Figure 1, A–C) was most likely the result of the conversion of CD27^–^ and CCR7^–^ cells into CD27^+^ and CCR7^+^ subsets. In addition, reprogramming further enhanced the expression of CD28 and TCF-1 in TCM and TTM cells (Figure 2A), while preventing the loss of CD127 and limiting the upregulation of T-bet upon stimulation of CD8^+^ T cell memory subsets (Figure 2B). Notably, reprogramming of TCM and TTM cells restrained the expression of granzyme B, IFN-γ, and IL-2, but TEM and TTE cells maintained these effector properties (Figure 2C). As was observed with bulk CD8^+^ T cells, all reprogrammed CD8^+^ T cell subsets showed increased TNF-α production (Figure 2C).

To better characterize the changes induced in reprogrammed CD8^+^ T cells, we performed gene expression analysis of sorted memory cells, analyzing 96 genes associated with CD8^+^ T cell function, differentiation, metabolism, and survival (see Supplemental Table 1). A principal component analysis (PCA) focused on TCM cells showed that reprogrammed and nonreprogrammed cells had a distinct gene expression profile (Figure 2D). In basal conditions, compared with nonreprogrammed cells, reprogrammed TCM cells exhibited lower expression levels of *IFNG*, *BATF*, *IFNGR1*, and *GZMK* genes, which are associated with an effector lineage (30, 31), as well as of *CD8A*, *CD244*, and *HAVCR2* (which encodes for TIM-3), which are associated with CD8^+^ T cell activation and exhaustion (ref. 32 and Figure 2E). In contrast, reprogrammed CD8^+^ T cells had a gene profile suggestive of metabolic quiescence (33), with lower levels of key metabolic regulators active during T cell activation, including *MLST8* (which encodes for the MTOR-associated protein LST8 homolog, required for mTORC pathway activation; ref. 34); *ESRRA* (which encodes for estrogen-related receptor α, a metabolic regulator of effector T cells; ref. 35); *GSL* and *GSL2* (which encode for glutaminase 1 and 2, respectively; ref. 36); and *PRKAA1* (which encodes for AMP-activated protein kinase, an important regulator of cell catabolic pathways; ref. 37 and Figure 2E). Importantly, after anti-CD3/anti-CD28 stimulation, reprogrammed TCM cells had higher expression levels of the antiapoptotic gene *BCL2* than did nonreprogrammed cells and lower expression levels of the effector-associated genes *FASLG*, *HAVCR2*, and *GZMB*, as well as *PRKAA1* (Figure 2E). The gene expression profile in reprogrammed TTM, TEM, and TTE cells was also modified by reprogramming (Supplemental Figure 2), and, among others, we detected higher expression of *BCL2* and the mTORC2-related gene *CDC42* in these memory subsets (Figure 2E, Supplemental Figure 2, and Supplemental Table 2), explaining in part the improved survival capacity of reprogrammed CD8^+^ T cells.

### The stem-like profile and polyfunctionality of reprogrammed CD8^+^ T cells is associated with the downregulation of anabolic metabolism and the mTORC1 pathway.

**Figure 3:**
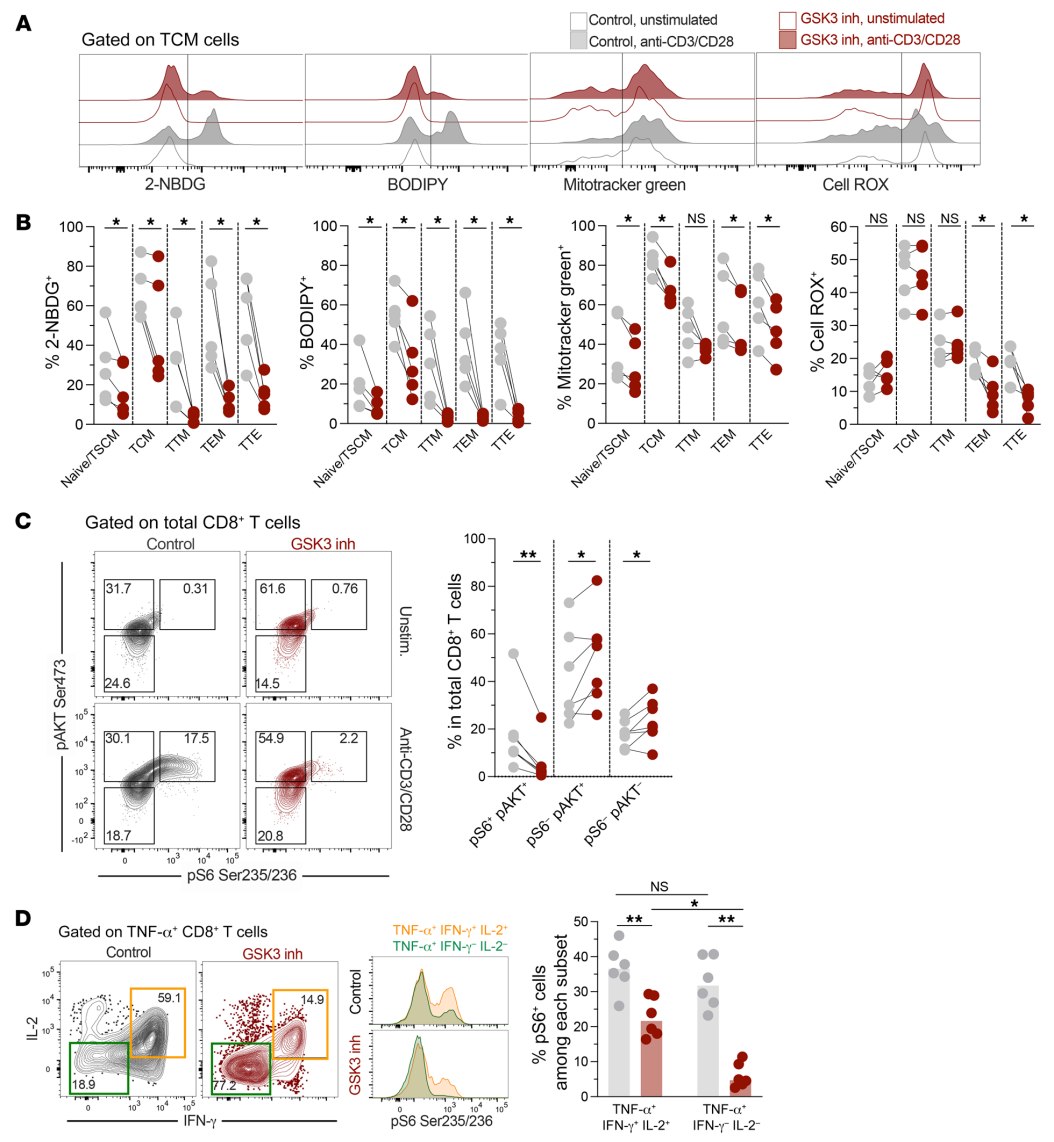
Modulation of anabolic metabolism in reprogrammed CD8^+^ T cells. (**A** and **B**) Total CD8^+^ T cells from individuals without HIV were treated with vehicle control or the GSK3 inhibitor, followed by incubation under basal conditions or with anti-CD3/anti-CD28 stimulation for 48 hours. (**A**) Analysis of the expression of 2-NBDG, BODIPY, MitoTracker Green, and CellROX among CD8^+^ T cell subsets. (**B**) Frequencies of 2-NBDG^+^, BODIPY^+^, MitoTracker Green^+^, and CellROX^+^ cells among CD8^+^ T cell subpopulations after stimulation (*n =* 5). (**C** and **D**) Total CD8^+^ T cells from individuals without HIV were treated with vehicle control or the GSK3 inhibitor, followed by incubation under basal conditions or with anti-CD3/anti-CD28 stimulation for 48 hours. (**C**) Flow cytometric analysis of the expression of p-S6 and p-AKT in total CD8^+^ T cells, and frequencies of p-S6^+^p-AKT^+^, p-S6^–^p-AKT^+^, and p-S6^–^p-AKT^–^ subsets in total CD8^+^ T cells (*n =* 7). (**D**) Flow cytometric analysis of IL-2 and IFN-γ expression among TNF-α^+^CD8^+^ T cells, after anti-CD3/anti-CD28 stimulation. Histograms show the expression of p-S6 in the indicated cell subsets, in reprogrammed and nonreprogrammed cells. Frequency of p-S6^+^ cells among TNF-α^+^IFN-γ^+^IL-2^+^ or TNF-α^+^IFN-γ^–^IL-2^–^ subsets (*n =* 6 individuals without HIV). *P < 0.05 and **P < 0.01, by Wilcoxon test (**B** and **C**) and Šidák’s multiple-comparison test (**D**). Data are from 2 independent experiments.

Considering that regulation of cell metabolism and mTORC pathways is a characteristic of memory-like CD8^+^ T cells (38), as we and others have reported for cells from HICs (8, 39), and that CD8^+^ T cell reprogramming readily modulated metabolism-related genes, we performed functional assays to evaluate the metabolic profile of reprogrammed cells. After anti-CD3/anti-CD28 stimulation, nonreprogrammed cells increased their 2-NBDG and BODIPY labeling (commonly used as indicators of glucose and lipid uptake) and augmented their mitochondrial mass and ROS production (Figure 3, A and B), a profile characteristic of recently activated T cells (33). However, reprogrammed CD8^+^ T cells showed lower levels of each of these metabolic parameters (Figure 3, A and B), consistent with reduced anabolic metabolism.

We next focused on the mTORC pathways and evaluated their activation by analyzing the phosphorylation of ribosomal S6 [p-S6 (Ser235/236)] and AKT [p-AKT (Ser473)] proteins, markers of activation of mTORC1 and mTORC2, respectively (40). Upon activation in control conditions, CD8^+^ T cells upregulated p-S6 in parallel with p-AKT (p-S6^+^p-AKT^+^ cells; Figure 3C). Consistent with the reported modulation of the mTORC1 pathway by GSK3 inhibitors (20), reprogrammed CD8^+^ T cells had less upregulation of p-S6 (Figure 3C). Notably, reprogrammed CD8^+^ T cells had higher p-S6^–^p-AKT^+^ levels (Figure 3C), confirming that the reprogrammed cells maintained a relative metabolic quiescence supported by preferential upregulation of the mTORC2 pathway. As shown above, reprogrammed CD8^+^ T cells had an increased capacity to produce TNF-α upon polyclonal stimulation. When analyzing IFN-γ and IL-2 production among TNF-α–producing CD8^+^ T cells, we identified 2 major subpopulations of cells: IFN-γ^+^IL-2^+^ and IFN-γ^–^IL-2^–^, the latter corresponding to cells producing TNF-α only and being increased among reprogrammed cells (Figure 3D). In keeping with our previous studies on cells from HICs (8), the production of TNF-α by reprogrammed cells was less dependent on mTORC1. This was particularly the case for cells only producing TNF-α, since the proportion of p-S6^+^ cells was lower in this subset relative to cells coproducing IFN-γ and IL-2 (Figure 3D). Altogether, these data indicate that the stem-like profile and polyfunctionality observed in reprogrammed CD8^+^ T cells are linked to the active regulation of anabolic metabolism, mTORC1 inhibition, and preferential engagement of mTORC2. Our data also highlight the differential role of mTORC pathways on the regulation of CD8^+^ T cell effector functions.

### Reprogramming of HIV-specific CD8^+^ T cells promotes polyfunctionality, survival, and expansion.

**Figure 4:**
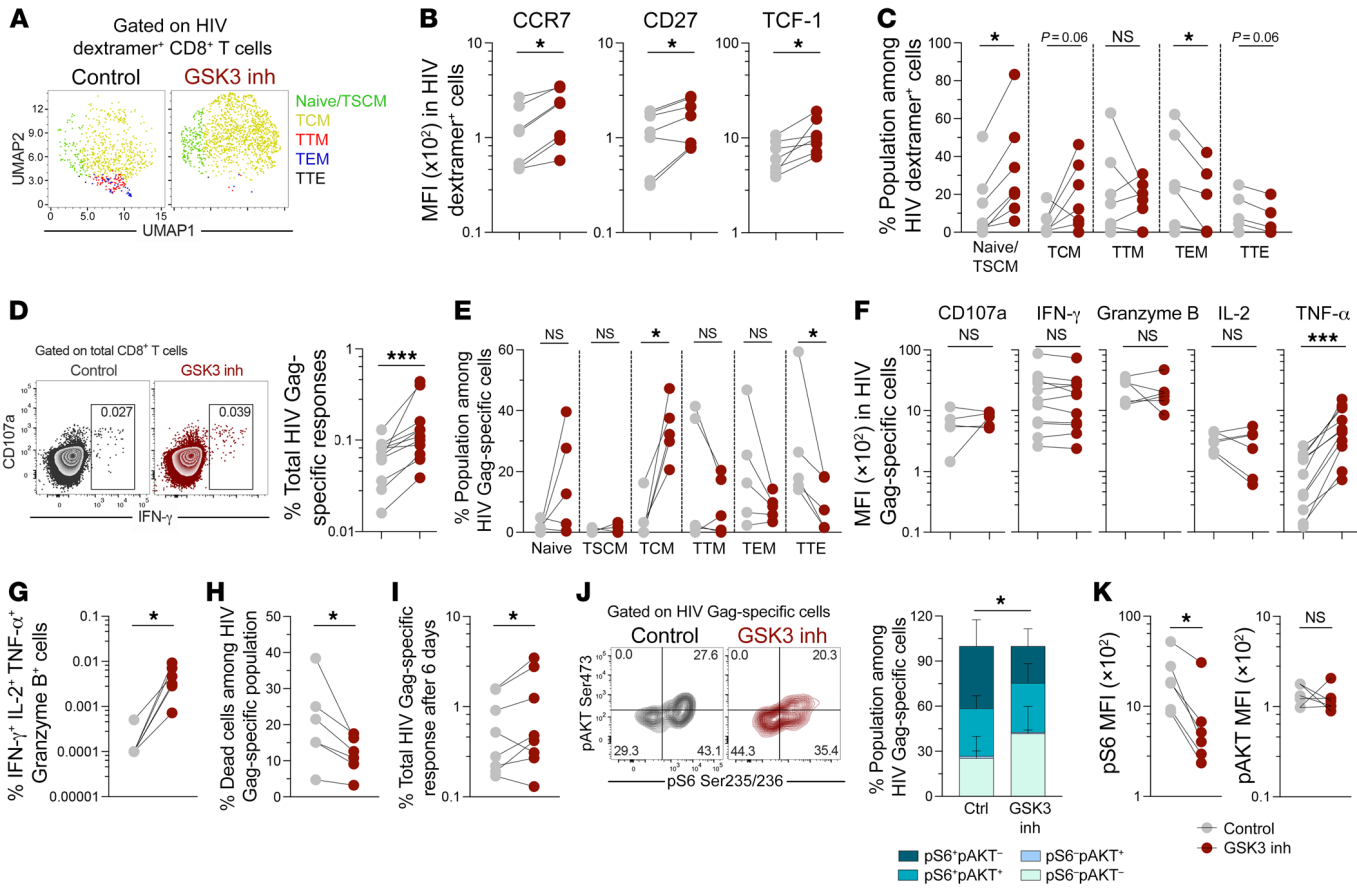
Enhanced functionality and survival of reprogrammed HIV-specific CD8^+^ T cells. After treatment with the GSK3 inhibitor, CD8^+^ T cells from people with HIV were stained with HLA-matched HIV dextramers for analysis of the phenotype of HIV dextramer^+^ cells. (**A**) UMAP plots generated from HIV dextramer^+^ CD8^+^ T cells after data concatenation (*n =* 4). Vehicle control and GSK3 inhibitor treatments as well as CD8^+^ T cell subpopulations were identified by manual gating and projected into the UMAP space. (**B**) Analysis of the expression of CCR7, CD27, and TCF-1 expression in HIV dextramer^+^ CD8^+^ T cells (*n =* 7). (**C**) Frequencies of CD8^+^ T cell subpopulations among HIV dextramer^+^ CD8^+^ T cells (*n =* 7). (**D**–**G**) After reprogramming, cells from people with HIV were stimulated for 6 hours with Gag peptides for analysis of the total frequency (IFN-γ^+^ or CD107a^+^ or IL-2^+^ or TNF-α^+^) of antigen-specific CD8^+^ T cells (*n =* 5) (**D**), the proportion of memory T cell subpopulations (*n =* 5) (**E**), the expression of CD107a, IFN-γ, granzyme B, IL-2, and TNF-α on a per-cell basis (*n =* 5–11) (**F**), and the frequency of polyfunctional cells (*n =* 6) (**G**). (**H** and **I**) Cells from people with HIV were stimulated for 6 days with Gag peptides and restimulated with the same peptides for another 12 hours, followed by analysis of the viability of proliferating HIV Gag–specific CD8^+^ T cells (*n =* 6) (**H**) and frequencies of the total (live IFN-γ^+^, IL-2^+^, or TNF-α^+^) HIV Gag–specific response (*n =* 8) (**I**). (**J** and **K**) After reprogramming, cells from people with HIV (*n =* 6) were stimulated for 6 hours with Gag peptides, followed by analysis of p-S6^+^p-AKT^–^, p-S6^+^p-AKT^+^, p-S6^–^p-AKT^+^, and p-S6^–^p-AKT^–^ cell subsets (**J**) and the intensity of expression of p-S6 and p-AKT (**K**) in HIV Gag–specific (IFN-γ^+^ and/or IL-2^+^) CD8^+^ T cells. *P < 0.05 and ***P < 0.001, by Wilcoxon test. Data are from 3 independent experiments.

Our results with polyclonally stimulated cells from people without HIV indicated that reprogramming improved several functional capacities of CD8^+^ T cells, without impairing others. However, HIV-specific CD8^+^ T cells from noncontrollers have a biased program characterized by exhaustion, low survival, and poor antiviral potential (8). Thus, we explored whether HIV-specific CD8^+^ T cells from noncontroller individuals (those on ART to suppress viremia; Supplemental Table 3) can also benefit from reprogramming and acquire properties found in HIV-specific CD8^+^ T cells associated with natural control of infection. First, we evaluated the effect of reprogramming on the phenotype of CD8^+^ T cells labeled with HIV-specific HLA dextramers. In line with the results for total CD8^+^ T cells from people without HIV, a uniform manifold approximation and projection (UMAP) analysis focused on HIV dextramer^+^ cells revealed enrichment of cell populations with a less-differentiated phenotype after reprogramming (Figure 4A), linked to the upregulation of CCR7, CD27, and TCF-1 (Figure 4B); an increase in the proportions of naive/TSCM and TCM cells; and a decrease in TEM and TTE cells (Figure 4C).

Next, we evaluated the functionality of HIV-specific CD8^+^ T cells, detected by the peptide-induced production of IFN-γ, IL-2, TNF-α, or by the expression of the degranulation marker CD107a. Reprogrammed CD8^+^ T cells contained a significantly higher frequency of total HIV-specific responses (Figure 4D) that were enriched in TCM cells (Figure 4E). Conversely, nonreprogrammed cells were mostly TEM and TTE cells (Figure 4E). Notably, we detected higher production of TNF-α by reprogrammed cells in terms of both frequency (Supplemental Figure 3A) and level of expression (Figure 4F) in HIV-specific CD8^+^ T cells, whereas there were no differences in the levels of CD107a, IFN-γ, granzyme B, or IL-2 (Figure 4F). We observed an overall increased polyfunctionality in reprogrammed HIV-specific CD8^+^ T cells relative to nonreprogrammed cells (Figure 4G). Of note, the survival capacity of reprogrammed HIV-specific cells was higher relative to that of nonreprogrammed cells (Figure 4H and Supplemental Figure 3B), in agreement with our observation that reprogramming induced the upregulation of antiapoptotic factors (Figure 2E). This resulted in enhanced survival of HIV-specific cells over 6 days of culturing in the setting of sequential peptide restimulation (Figure 4I).

We next wondered whether the effects of CD8^+^ T cell reprogramming are restricted to HIV-specific cells or also affect other antigen-specific cells, such as those specific to human cytomegalovirus (HCMV). Our analysis of HCMV-specific CD8^+^ T cells from people without HIV did not reveal an increase in the total frequency of HCMV-specific CD8^+^ T cells after reprogramming (Supplemental Figure 4, A and B). Nonetheless, reprogrammed HCMV-specific were enriched in a TCM phenotype (Supplemental Figure 4C) and had higher TNF-α expression relative to that in nonreprogrammed cells (Supplemental Figure 4D). We next performed parallel stimulations with HCMV pp65 or HIV Gag peptides and evaluated the phenotype and function of antigen-specific cells from the same individuals with HIV. In line with our previous results (Figure 4F), we did not observe significant differences in the frequencies of CD107a^+^ or IFN-γ^+^ HCMV or HIV-specific CD8^+^ T cells between reprogrammed versus nonreprogrammed cells (Supplemental Figure 4E). Instead, we readily observed an increase of TNF-α^+^ HCMV and HIV-specific CD8^+^ T cells in reprogrammed cells relative to nonreprogrammed cells. The enhancement appeared stronger for HIV-specific cells, since the Δ change in TNF-α production by reprogrammed/nonreprogrammed cells was higher in HIV-specific than in HCMV-specific cells (Supplemental Figure 4F). Overall, these data suggest that the effects of reprogramming might be stronger in HIV-specific cells than in HCMV-specific cells, which may be related to metabolic and functional differences in these antigen-specific cell populations in the same host (8, 41).

Collectively, these results indicate that reprogramming of HIV-specific CD8^+^ T cells from noncontrollers promoted a quantitatively and qualitatively superior response to antigen stimulation, reflected in higher functionality, survival, and expansion capacity, which are features observed in cells from natural HIV-1 controllers.

### Metabolic plasticity is restored in reprogrammed HIV-specific CD8^+^ T cells.

We next assessed whether reprogramming could improve the marked dependency on mTORC1 and glycolysis that characterizes HIV-specific CD8^+^ T cells from HIV-1 noncontrollers (8). Stimulation of nonreprogrammed cells from noncontrollers with HIV-1 Gag peptides resulted in the upregulation of p-S6 (Supplemental Figure 5A), both alone and together with p-AKT (Figure 4J). In contrast, reprogrammed HIV-specific CD8^+^ T cells had lower proportions of p-S6^+^p-AKT^–^ cells (Figure 4K). We also detected significantly lower expression of p-S6 on a per-cell basis in reprogrammed HIV-specific CD8^+^ T cells (Figure 4K and Supplemental Figure 5A). In contrast, we did not observe significant differences in the intensity of p-AKT in reprogrammed cells (Figure 4, J and K). These data indicate that reprogrammed HIV-specific CD8^+^ T cells had a diminished dependency on mTORC1 for supporting a strong antigen-induced response, while preserving mTORC2 activation to exert their functions. We additionally evaluated glucose dependency of reprogrammed HIV-specific CD8^+^ T cells in an assay in which Gag peptide stimulation was performed in medium with and without glucose (8). Glucose deprivation decreased the frequency of total HIV-specific cells at comparable levels in reprogrammed versus nonreprogrammed cells (Supplemental Figure 5, B and C). However, reprogrammed cells maintained higher production of TNF-α despite glucose deprivation (Supplemental Figure 5D). Altogether, these data indicate that reprogramming of HIV-specific cells from noncontrollers promotes metabolic plasticity and decreases metabolic restrictions.

### Reprogramming reinvigorates CD8^+^ T cells to suppress HIV-1 replication.

**Figure 5:**
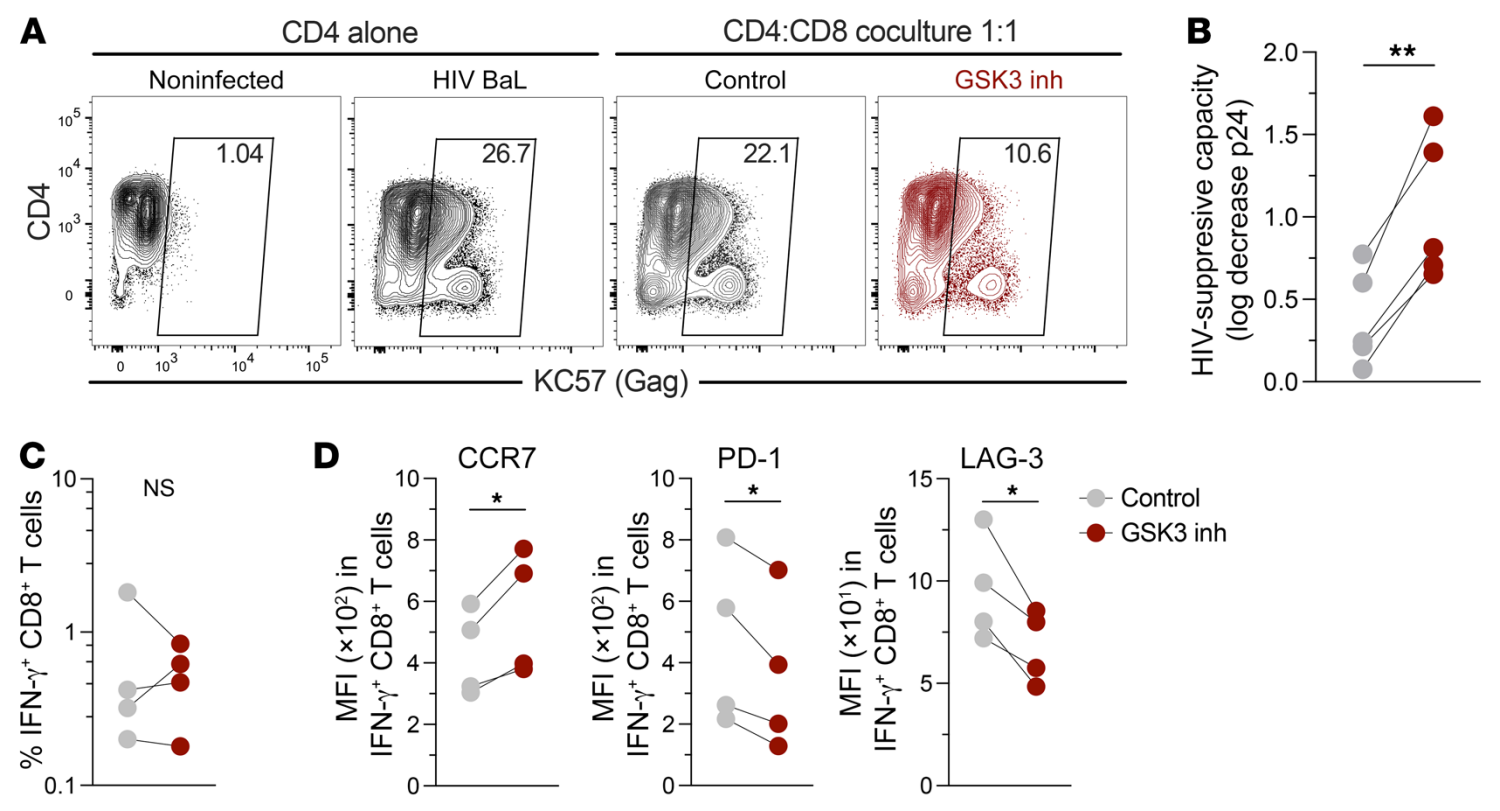
Reprogramming increases the HIV-1–suppressive capacity of CD8^+^ T cells. HIV-1 BaL–superinfected CD4^+^ T cells from people with HIV were cultured alone or in the presence of autologous nonreprogrammed or reprogrammed CD8^+^ T cells. After 7 days, the levels of infection were measured by flow cytometry (KC57 anti-Gag antibody) or ELISA (p24 in culture supernatant). (**A**) Representative flow cytometric analysis of the frequency of infected CD4^+^ T cells (from a total of 4 donors). (**B**) HIV-suppressive capacity of nonreprogrammed and reprogrammed CD8^+^ T cells (log_10_ decrease of p24 levels in culture supernatant; *n =* 5 individuals, with the median of triplicates for each experiment). (**C** and **D**) The frequency of IFN-γ^+^ HIV-specific CD8^+^ T cells (**C**) and expression of CCR7, PD-1, and LAG-3 in HIV-specific CD8^+^ T cells (**D**) were measured after 7 days of coculturing. *P < 0.05 and **P < 0.01, by Wilcoxon test.

To further elucidate the antiviral potential of reprogrammed CD8^+^ T cells, we evaluated the capacity of nonstimulated cells from noncontroller individuals under ART to suppress HIV-1 infection of autologous CD4^+^ T cells (42). According to our previous studies (8, 10, 43), nonreprogrammed CD8^+^ T cells from HIV-1 noncontrollers on ART had a poor capacity to counteract HIV-1 infection when cocultured with infected CD4^+^ T cells; in contrast, reprogramming strongly improved the capacity of CD8^+^ T cells to suppress HIV-1 infection (Figure 5, A and B, and Supplemental Figure 6A). It should be noted that we cannot exclude the fact that, at the start of the coculture, the frequency of HIV-specific CD8^+^ T cells was higher upon reprogramming because of the promotion of the survival capacity of the cells. However, we did not observe differences in the frequency of IFN-γ^+^CD8^+^ T cells (presumably HIV-specific) between reprogrammed and nonreprogrammed cells at the end of the coculture (day 7; Figure 5C), but reprogrammed IFN-γ^+^ CD8^+^ T cells maintained a memory-like profile, with higher expression of CCR7 and decreased expression of the inhibitory receptors PD-1 and LAG-3 (Figure 5D and Supplemental Figure 6B). Therefore, our data indicate that reprogramming of CD8^+^ T cells from noncontrollers strengthens their direct anti-HIV potential, while restricting the acquisition of a terminally differentiated, exhausted program.

### Reprogrammed CD8^+^ T cells respond better to homeostatic γ-chain cytokines.

**Figure 6:**
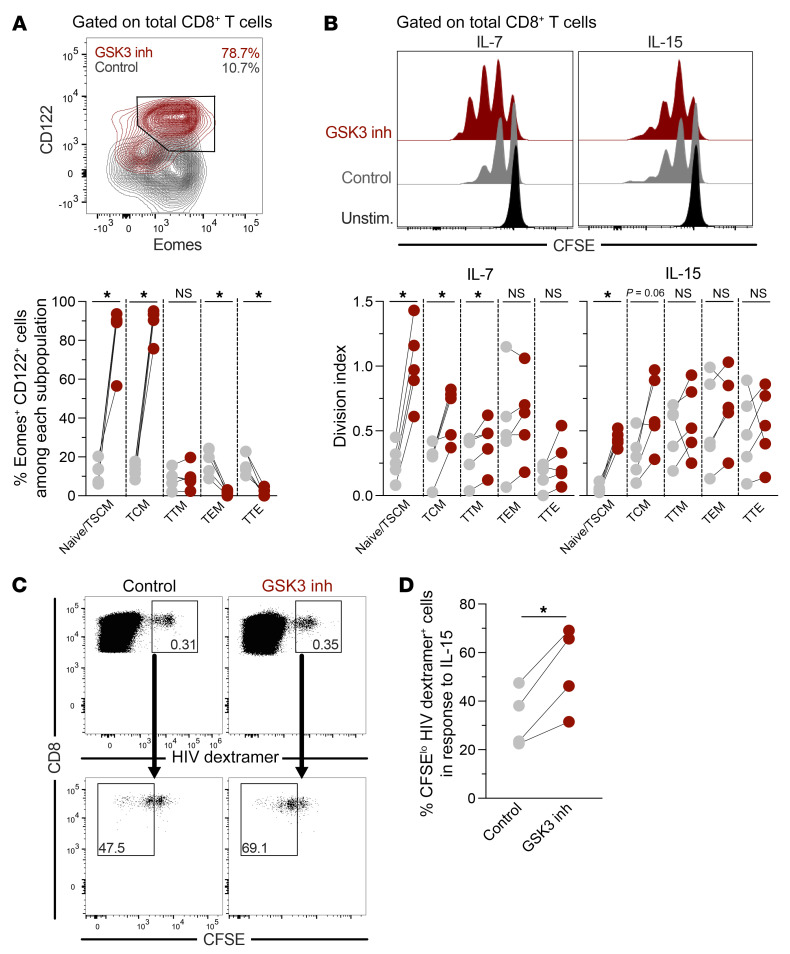
Superior response to γ-chain cytokines by reprogrammed HIV-specific CD8^+^ T cells. (**A**) Total CD8^+^ T cells from people without HIV were treated with vehicle control or the GSK3 inhibitor, followed by evaluation of Eomes and CD122 expression in CD8^+^ T cell subsets (*n =* 5). (**B**) After vehicle control or GSK3 inhibitor treatment, CD8^+^ T cells from people without HIV were left unstimulated or stimulated with IL-7 or IL-15 for 6 days, followed by analysis of cell proliferation (*n =* 5; data are from 2 independent experiments). (**C** and **D**) CD8^+^ T cells from people with HIV were treated with vehicle control or the GSK3 inhibitor, followed by stimulation with IL-15 for 6 days, for analysis of the proliferation of HIV dextramer^+^ CD8^+^ T cells (*n =* 4; data from 3 independent experiments). *P < 0.05, by Wilcoxon test.

The long-term maintenance of memory T cells requires an optimal response to the γ-chain cytokines IL-7 and IL-15 for self-renewal (44). Thus, we evaluated how reprogramming affected the ability of CD8^+^ T cells to respond to these homeostatic cytokines. We found that reprogrammed bulk CD8^+^ T cells were characterized by higher levels of CD127 (IL-7Rα chain; Figure 1E). We also found that reprogrammed CD8^+^ T cells had higher expression of CD122 (IL-2Rβ chain, a component of the IL-15 receptor) and the IL-15Rα chain CD215 (Supplemental Figure 7, A and B), as well as a higher proportion of cells coexpressing the transcription factor eomesodermin (Eomes) and CD122 (Figure 6A). This may be related to previous observations showing that TCF-1 regulates the expression of Eomes, which in turn promotes CD122 (24).

The increased expression of IL-7 and IL-15 receptors on reprogrammed bulk CD8^+^ T cells from people without HIV was reflected in augmented proliferation in response to both cytokines when compared with nonreprogrammed cells (Figure 6B). As expected, the proliferative potential was higher in naive/TSCM and TCM cells than in more differentiated cells (Figure 6B). Reprogramming also improved the proliferative response to IL-15 of HIV dextramer^+^ cells from HIV-1 noncontrollers (Figure 6, C and D). Finally, the maturation of the effector response induced by IL-15 in terms of upregulation of T-bet was comparable between reprogrammed and nonreprogrammed cells (Supplemental Figure 7C). Overall, our results show that CD8^+^ T cell reprogramming improves the response to homeostatic γ-chain cytokines in cells with otherwise diminished capacity.

## Discussion

We report that reprogramming of HIV-specific CD8^+^ T cells from HIV-1 noncontrollers via GSK3 inhibition confers higher polyfunctionality, survival, expansion capacity, diminished dependency on mTORC1 and glucose, a better responsiveness to γ-chain cytokines, and a strong HIV-1–suppressive capacity. These characteristics have been previously associated with efficient responses in natural HIV-1 controllers (4–8). Thus, such CD8^+^ T cell reprogramming represents a promising option to enhance the efficacy of cell-based therapies for HIV-1 infection.

Our choice of targeting GSK3 was based on the implication of this molecule in the modulation of several intracellular pathways that we have identified to be of potential relevance in virus-specific CD8^+^ T cells from HIV-1 (8) and SIV natural controllers (9). BIO and TWS119, the 2 molecules that we used here, are highly selective inhibitors of GSK3 and induce activation of the Wnt/β-catenin/TCF-1 pathway (45). Higher expression of TCF-1 is a characteristic of virus-specific CD8^+^ T cells in HIV-1 and SIV controllers (7, 9, 16), and the intensification of its function promotes CD8^+^ T cell stemness, which associates with potent virus and tumor control (7, 46). It is also well described that stem-like memory TCF-1^+^ CD8^+^ T cells have strong antiviral potential and therapeutic benefit (13–15, 47, 48), related to their longevity and enhanced ability to expand and differentiate into effector subsets (48, 49). In addition, GSK3 interacts with the mTOR signaling network (50), and its inhibition promotes activation of the mTORC2 pathway (51). The mTORC2 pathway promotes the generation of memory CD8^+^ T cells (52) and is preferentially upregulated in HIV-specific CD8^+^ T cells from HICs (8).

In line with the effects of GSK3 inhibition, our study shows that CD8^+^ T cell reprogramming promoted the expression of TCF-1, a TCM and TSCM phenotype, as well as restrained effector differentiation. Reprogrammed CD8^+^ T cells also regulated proliferation to maintain quiescence and to limit the consequences of excessive activation (21, 53, 54). Correspondingly, reprogrammed CD8^+^ T cells shifted their transcriptomic profile toward a program of quiescence and survival and downregulated several genes associated with anabolic metabolism (55), as reflected in lower glucose and lipid consumption, and mitochondrial activity. These data are in line with previous studies showing the critical role of metabolism in the CD8^+^ T cell effector versus memory fate (56, 57) and the quiescent profile of less differentiated subsets (49, 58). Remarkably, both polyclonal and HIV-specific reprogrammed CD8^+^ T cells showed lower activation of mTORC1 and a predilection for mTORC2 pathway activation to sustain their activities. Moreover, reprogrammed HIV-specific CD8^+^ T cells depended less on glucose. Such metabolic plasticity is also found in cells from HICs and may be particularly helpful in hostile metabolic environments such as in tissues, where infected cells compete for nutrients (59), and where a high antigen load and inflammation affect the survival and function of CD8^+^ T cells (60). The modulation of metabolism and the induction of a stem-like profile are likely related and synergize to improve CD8^+^ T cell antiviral potential, even in settings of persistent or strong antigen stimulation. Indeed, the maintenance of a stem-like profile and less effector differentiation and immune exhaustion were associated with stronger HIV-1 suppression by reprogrammed CD8^+^ T cells. These properties exhibited by reprogrammed HIV-specific CD8^+^ T cells might contribute to long-term viral control in vivo. Supporting this notion, stem-like, SIV-specific CD8^+^ T cells are maintained over the long term and are linked to the natural control of infection (9).

We found that reprogramming did not affect all CD8^+^ T cells uniformly. Although BIO induced very similar effects globally on CD8^+^ T cells, reprogramming seemed to have the strongest enhancing effect on the activities of HIV-specific CD8^+^ T cells from noncontrollers when compared with cells responding to polyclonal stimulation or HCMV peptides. We have previously shown that HCMV and HIV-specific cells from HICs are relatively similar in terms of memory metabolic program (8). In contrast, HIV-specific cells from noncontrollers have an effector-like and exhausted profile (8, 61, 62), and such a biased program can be found even among the TCM population (8). These divergent signatures in cells from controllers and noncontrollers could be related to the TCR signaling and priming signals they received in vivo early after infection (9), which may be exacerbated by the persistence of high amounts of antigen and inflammation in noncontroller individuals. Thus, it was expected that HIV-specific CD8^+^ T cells derived from noncontrollers benefited the most from the effects of reprogramming when compared with better-fitted HCMV-specific cells. Along these lines, our results show that the skewed program of HIV-specific CD8^+^ T cells from noncontrollers was at least partially reversible.

Our results also show that not all CD8^+^ T cells activities were equally regulated by metabolic pathways. Observations in one of our previous studies suggested that polyfunctionality, and in particular TNF-α production, might depend on the mTORC2 pathway (8). This aspect was confirmed here. We observed a selective increase in the production of TNF-α by reprogrammed CD8^+^ T cells, which was related to the inhibition of mTORC1, preservation of the mTORC2 pathway, and less dependency on glycolysis. In contrast, IFN-γ and IL-2 production was associated with mTORC1 activation. In keeping with our results, previous studies have demonstrated that TNF-α production is associated with a less-differentiated CD8^+^ T cell profile (63, 64), whereas the transcription factor NF-κB (regulator of TNF-α expression) is active in stem-like CD8^+^ T cells (48, 65), suggesting a role of this signaling pathway as a regulator of this cell subset. The underlying mechanism of greater TNF-α production by reprogrammed CD8^+^ T cells might be related to epigenetic modifications and/or posttranscriptional events (64, 66) that deserve further study. Moreover, although further studies are necessary to fully elucidate this aspect, our data suggest that the production of TNF-α in the absence of mTORC1 activity (p-S6 expression) could serve as an additional marker of stem-like memory CD8^+^ T cells. In the context of HIV-1 control, TNF-α might help to inhibit HIV replication, as has previously been shown in monocytes and macrophages (67, 68), and its production, together with other soluble factors, may contribute to establishing an antiviral environment while CD8^+^ T cells are arrested on killed target cells (69). Interestingly, a previous study showed that the cytotoxic capacity of HIV-specific CD8^+^ T cells is enhanced in cells producing both IFN-γ and TNF-α (70). However, this did not appear to be related to a direct cytotoxic effect of TNF-α. Simultaneous production of IFN-γ and TNF-α, rather, revealed a subset of cells with qualitatively superior cytotoxic potential (70). Moreover, TNF-α has also been shown to reverse the latency of provirus in persistently infected cells (71, 72). This might be relevant in vivo, where local production of TNF-α may reveal the presence of latently infected cells, facilitating their elimination by the cytotoxic activity of the CD8^+^ T cells. Thus, TNF-α production and polyfunctionality are features of the stem-like profile in HIV-specific CD8^+^ T cells promoted by reprogramming, that, together with other functional attributes (including greater survival, proliferative potential, and metabolic plasticity) may collectively contribute to the augmented antiviral potential observed.

We propose that reprogramming HIV-specific CD8^+^ T cells may be of potential interest for interventions aimed at HIV-1 remission. Two beneficial effects could be expected: first, we have shown here that reprogramming reinvigorates the antiviral potential of HIV-specific CD8^+^ T cells; second, the promotion of self-renewal and long-term survival of the cells may improve the therapeutic efficacy of adoptive immunotherapies. Indeed, one of the major caveats in adoptive immunotherapies is the low persistence of transferred cells, which impairs the long-term therapeutic efficacy, as has been observed in HIV-1^+^ individuals receiving autologous, ex vivo–expanded, HIV-specific CD8^+^ T cells (73–75). In line with this notion, complete cancer regression and persistence of transferred tumor-reactive CD8^+^ T cells have been associated with a stem-like cell population capable of self-renewal, expansion, and antitumor responses (76). Of note, CD8^+^ T cell reprogramming could be combined with other interventions to further enhance their antiviral potential. We have previously shown that short-term IL-15 treatment of HIV-specific CD8^+^ T cells from noncontrollers increased the capacity of these cells to mobilize mitochondrial activities and suppress HIV-1 infection of CD4^+^ T cells (8). As reprogrammed CD8^+^ T cells are more responsive to IL-15 in terms of proliferation and maturation of their effector profile, we hypothesize that a combined approach may have synergistic effects.

A limitation of our study is that all analyses were performed ex vivo. Therefore, further study is needed to elucidate the antiviral potential and lifespan of adoptively transferred, reprogrammed HIV-specific CD8^+^ T cells in vivo, considering potential escape mutations, inflammatory conditions, and the nature of the HIV-1 reservoir. Indeed, a high proportion of latent viruses have escape mutations to evade the CD8^+^ T cell response (77), which may compromise the efficacy of CD8^+^ T cell–based cure strategies. However, the superior in vivo antitumor response of transferred stem-like CD8^+^ T cells in human cancer (76), as well as the enhanced anti-HIV potential exhibited by reprogrammed CD8^+^ T cells ex vivo, support the rationale for exploring this immunotherapy, in combination with other methodologies (such as γ-chain cytokines or chimeric antigen receptor cells), in the context of HIV-1 infection. Another limitation to our study is that we only evaluated the impact of reprogramming in CD8^+^ T cells from individuals with HIV receiving ART, and not from viremic untreated individuals, who are expected to have more dysfunctional cells. However, individuals on ART would be the main target population for these immunotherapies, given the well-known benefits of treatment initiation. Moreover, in this study, we only analyzed cells derived from blood and not from tissues. Thus, future studies could evaluate whether GSK3 inhibitors also modify the program of tissue-resident memory cells. Furthermore, in our study, we propose the use of reprogramming with BIO for adoptive cell therapies. Given the multisystemic effects that GSK3-targeting drugs could have in vivo (78), it will be necessary to explore the use of other GSK3 inhibitors with proven clinical safety and tolerability (78, 79) that could be injected systemically to reprogram CD8^+^ T cells in vivo.

In summary, here we show that HIV-specific CD8^+^ T cell pharmacologic reprogramming into stem-like cells conferred several properties associated with natural control of infection and helped to improve the response to immunomodulators. CD8^+^ T cell reprogramming could be used to potentiate the therapeutic efficacy of this cell population in adoptive transfer strategies as well as the effect of other immunotherapies, in the search for an HIV-1 cure or remission or in the development of therapeutic vaccines. In addition, the benefits of T cell reprogramming could be extended to the context of other chronic viral infections or tumors in which antigen-specific CD8^+^ T cells have a skewed and dysfunctional profile.

## Methods

### Participants.

Samples from people without HIV were obtained from the Établissement Français du Sang in the context of a collaboration agreement with the Institut Pasteur. HIV-1 noncontroller participants (on ART for at least 2 years and having undetectable HIV-1 RNA) were recruited in the context of the ANRS EP36 XII mTOR study or the ANRS CO6 PRIMO cohort. Clinical data for participants included are summarized in Supplemental Table 3. All analyses were conducted on cryopreserved PBMCs isolated from blood samples.

### In vitro reprogramming of CD8^+^ T cells.

Frozen PBMCs were thawed and rested overnight at 37°C, 5% CO_2_ in RPMI 1640 GlutaMAX medium (Thermo Fisher Scientific) supplemented with 10% fetal calf serum and penicillin/streptomycin (complete medium). For reprogramming of CD8^+^ T cells followed by polyclonal stimulation, cells were isolated by negative magnetic bead sorting (STEMCELL Technologies). For reprogramming of CD8^+^ T cells followed by antigen-specific stimulation, we used PBMCs and magnetically separated CD8^+^ T cells and non-CD8^+^ T cells (REAlease CD8 MicroBead Kit; Miltenyi Biotec). Then, CD8^+^ T cells were treated with the GSK3 inhibitor BIO (MilliporeSigma), at 3 μM, or an equivalent concentration of dimethyl sulfoxide (DMSO) vehicle control, in complete medium, for 12 hours at 37°C, 5% CO_2_. Non-CD8 cells were left resting during this time in complete medium. CD8^+^ T cells were washed with complete medium and stimulated with the peptide pool in the presence of non-CD8^+^ T cells. To confirm the effects of BIO, another GSK3 inhibitor, TWS119 (MilliporeSigma), was used under similar conditions (at 3 μM, 12-hour incubation).

### Polyclonal, antigen-specific, and cytokine stimulation of CD8^+^ T cells.

After reprogramming, CD8^+^ T cells were polyclonally stimulated with plate-bound anti-CD3 and anti-CD28 antibodies (both at 1 μg/mL; clones OKT3 and CD28.2, respectively; both from Thermo Fisher Scientific), in the presence or absence of human recombinant ICAM-1/CD54 Fc Chimera Protein (at 50 μg/mL; R&D Systems) and cultured for 48 hours at 37°C, 5% CO_2_, accordingly. For intracellular cytokine analyses upon polyclonal stimulation, brefeldin A (at 10 μg/mL; MilliporeSigma) was added to the cells and incubated during the last 12 hours of culturing. Antigen-specific stimulation was performed by mixing CD8^+^ and non-CD8 cells (maintaining the original cell ratio) and incubating with overlapping peptide pools encompassing HIV-1 consensus subtype B Gag or HCMV pp65 (both at 2 μg/mL; obtained through the NIH AIDS Reagent Program, Division of AIDS, National Institute of Allergy and Infectious Diseases (NIAID), NIH, catalog numbers 12425 and 11549). For subsequent intracellular cytokine analysis, cells were incubated for 6 hours in the presence of anti-CD28^+^CD49d antibodies (clones L293 and L25, respectively; both at 1 μg/mL; BD Biosciences) and an anti–CD107a FITC antibody (BD Biosciences) plus brefeldin A (at 10 μg/mL) and monensin (at 1 μg/mL; BD Biosciences), with the 2 latter added 30 minutes after the start of all incubations. In some experiments, cells were cultured in RPMI medium without glucose (MP Biomedicals). CD8^+^ T cell proliferation was evaluated by CFSE dilution (at 1 μM, Thermo Fisher Scientific). In additional experiments, reprogrammed CD8^+^ T cells were stimulated with IL-7 or IL-15 (both at 10 ng/mL; R&D Systems) and cultured for 6 days. In all cases, the cells were cultured at a density of 2 × 10^6^ cells/mL.

### Flow cytometric analysis.

After culturing, cells were stained with the LIVE/DEAD Fixable Aqua Dead Cell Stain kit (Thermo Fisher Scientific) along with anti–CD3 Alexa Fluor 700 or APC eFluor 780 and anti–CD8 PE Texas Red antibodies. For phenotype analyses, cells were additionally stained with anti–CCR7 PE Cy7, anti–CD45RA APC H7, anti–CD27 PerCP Cy5.5, anti-CD95 APC, or anti–CD127 Alexa Fluor 488 antibodies, followed by incubation for 15 minutes at room temperature. In some experiments, cells were stained for 10 minutes at room temperature with APC-conjugated dextramers (Immudex): HLA-A*0201 (SLYNTVATL) Gag, HLA-A*0301 (RLRPGGKKK) Gag, HLA-A*0301 (QVPLRPMTYK) Nef, and HLA-B*2705 (KRWIILGLNK) Gag-Pol. Additional surface staining panels included anti–HLA-DR Superbright 780, anti–CD38 Superbright 600, anti–PD-1 BV421, anti–LAG-3 APC eFluor 780, anti–TIM-3 PE Cy7, and anti–TIGIT BV786 antibodies, or anti–CD122 PE and anti–CD215 FITC antibodies. The BD Transcription Factor Buffer Set (BD Biosciences) was used for cell fixation and permeabilization. Intracellular staining panels for the detection of transcription factors included anti–TCF-1 PE, anti–T-bet V450, anti–Eomes APC, anti–TOX efluor 660, anti–BLIMP-1 CF594, or anti–BCL6 Alexa Fluor 488. Intracellular cytokine staining panels included anti–IFN-γ PE Cy7 or V450, anti–IL-2 APC-R700, anti–TNF-α PerCP Cy5.5, or anti–granzyme B Alexa Fluor 647 antibodies. In some experiments, cells were fixed and permeabilized with Phosflow Fix and Perm buffers (BD Biosciences), and cells were stained with anti–p-S6 (Ser235/236) Pacific blue and anti–p-AKT (Ser473) FITC antibodies (Cell Signaling Technology). Cell acquisition was performed using an LSR II, LSRFortessa X-20, or ARIA III flow cytometer (all from BD Biosciences), and data were analyzed with FlowJo software (version 10, BD Biosciences). The list of flow cytometry antibodies used in this study is provided in Supplemental Table 4.

### Measurement of metabolite uptake.

CD8^+^ T cells were split into 4 parts and put into contact with 2-NBDG [2-(*N*-(7-nitrobenz-2-oxa-1,3-diazol-4-yl)amino)-2-deoxyglucose] (150 μM, 30 min) for glucose uptake measurement; BODIPY 500/510 C_1_, C_12_ (4,4-difluoro-5-methyl-4-bora-3a,4a-diaza-*s*-indacene-3-dodecanoic acid) (5 μM, 5 min) for fatty acid uptake measurement; MitoTracker Green FM (100 nM, 45 min) for mitochondrial mass measurement; and CellROX Deep Red (5 μM, 30 min) for ROS measurement (all from Thermo Fisher Scientific). After these incubations, cells were stained with the LIVE/DEAD Fixable Aqua Dead Cell Stain kit as well as with phenotype antibodies, as described above.

### Gene expression analysis in sorted memory cells.

Purified CD8^+^ T cells were stained with the LIVE/DEAD Fixable Aqua Dead Cell Stain kit and the following antibodies: anti–CD3 Alexa Fluor 700, anti–CD8 APC Cy7, anti–CCR7 PE Cy7, anti–CD45RA BV421, and anti–CD27 PE (all from BD Biosciences). Viable central memory, transitional memory, effector memory, and terminal effector CD8^+^ T cells were bulk sorted using the BD FACSAria III (BD Biosciences) and left to rest for at least 6 hours in complete medium before treatment with the GSK3 inhibitor or vehicle control. Then, cells were washed and stimulated with plate-bound anti-CD3/anti-CD28 antibodies (1 μg/mL) for 48 hours. After culturing, cells were counted, and a total of 2500 cells per condition were placed in 96-well plates containing VILO Reaction Mix, SUPERase-In, and NP40 (all from Thermo Fisher Scientific). Plates were snap-frozen and stored at –80°C. Analysis of gene expression was performed as previously described (8), using Delta Gene primers, 96.96 Dynamic Array chips, and a Biomark instrument for microfluidics-based quantitative PCR (Fluidigm). Linear derivative mode baseline correction was applied. Data were normalized to *GAPDH* as the housekeeping gene, and the ΔCt method was applied. Gene expression values are plotted as log 2^–ΔCt^.

### Viral suppression assays.

PBMCs from individuals with HIV were used to evaluate the capacity of reprogrammed CD8^+^ T cells to suppress HIV infection ex vivo (42). CD4^+^ and CD8^+^ T cells were separated by, respectively, successive positive and negative magnetic bead sorting (STEMCELL Technologies). Human CD4^+^ T cells were cultured for 3 days in complete medium supplemented with IL-2 (100 IU/mL), in the presence of phytohemagglutinin (PHA) (2 μg/mL). In parallel, purified CD8^+^ T cells were treated with DMSO vehicle control or the GSK3 inhibitor for 12 hours, followed by washing and incubation for 6 days in complete medium alone. Then, activated CD4^+^ T cells were superinfected with HIV-1 BaL strain by spinoculation for 1 hour at 2000*g* and 22°C and cultured alone or with CD8^+^ T cells at a 1:1 ratio for 7 days in complete medium supplemented with IL-2. Virus replication was measured according to p24 production in the culture supernatants using an ELISA (XpressBio) or by flow cytometry by quantifying intracellular HIV-1 Gag products (KC57-FITC antibody; Beckman Coulter). The HIV-suppressive capacity of CD8^+^ T cells was calculated as the log_10_ fold decrease in the median levels of p24 when CD4^+^ T cells were incubated in the presence of CD8^+^ T cells. For the detection of HIV-specific CD8^+^ T cells, at the end of coculturing and previous collection of supernatants, cells were incubated with brefeldin A (10 μg/mL), monensin (2 μg/mL), and an anti–CD107a BV786 antibody (BD Biosciences), followed by intracellular cytokine staining, as described above.

### Statistics.

GraphPad Prism, version 9.0 (GraphPad Software), was used for statistical analysis. Data are presented as medians and ranges. The Wilcoxon test was used for the comparison of 2 paired data. Friedman’s test and Dunn’s multiple-comparison test were used for the comparison of 3 or more paired groups. Šidák’s method was applied to correct for multiple comparisons where needed. All *P* values of less than 0.05 were considered statistically significant. To determine differential expression of genes for each CD8^+^ T cell memory population, we defined a mixed-effect model that included treatment (vehicle control versus GSK3 inhibitor) and condition (unstimulated or anti-CD3/anti-CD28 stimulated) and their interaction as fixed effects, and considered participant and technical replicates as random effects. The parameters were estimated using the lme4 package (version 1.1-23) in R.

### Study approval.

This study was approved by the ethics committee (Comité de Protection des Personnes) of Île-de-France XI. All study participants provided informed consent.

## Author contributions

FPC and ASC conceived the study. FPC, CP, and VM performed experiments and analyzed the data. SV assisted with computational analyses. FB, PDT, MM, KB, LW, CJ, CB, CG, LM, and OL helped with participant recruitment. FPC and ASC wrote the manuscript. All authors revised the manuscript. ASC provided financial support and supervised the work.

## Supplementary Material

Supplemental data

## Version 1 04/05/2022

In-Press Preview

## Version 2 06/01/2022

Electronic publication
