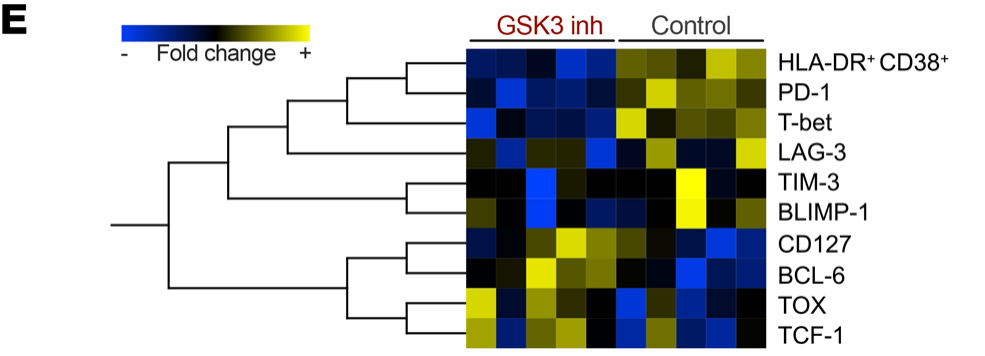# Reprogramming dysfunctional CD8^+^ T cells to promote properties associated with natural HIV control

**DOI:** 10.1172/JCI167843

**Published:** 2023-01-17

**Authors:** Federico Perdomo-Celis, Caroline Passaes, Valérie Monceaux, Stevenn Volant, Faroudy Boufassa, Pierre de Truchis, Morgane Marcou, Katia Bourdic, Laurence Weiss, Corinne Jung, Christine Bourgeois, Cécile Goujard, Laurence Meyer, Michaela Müller-Trutwin, Olivier Lambotte, Asier Sáez-Cirión

Original citation: *J Clin Invest*. 2022;132(11):e157549. https://doi.org/10.1172/JCI157549

Citation for this corrigendum: *J Clin Invest*. 2023;133(2):e167843. https://doi.org/10.1172/JCI167843

In Figure 1E, the data for BCL-6 was inadvertently omitted, with a duplicate of the data for TOX shown on that line instead. The PDF and HTML versions have been updated, and the correct figure panel is below.

The authors regret the error.

## Figures and Tables

**Figure 1E.**