# Supplementary material for: Reprogramming dysfunctional CD8+ T cells to promote properties associated with natural HIV control
Source: J Clin Invest. 2022 Jun 1;132(11):e157549. doi: 10.1172/JCI157549 (PMC9151687; doi:10.1172/JCI157549)
Supplement: Supplemental data [file jci-132-157549-s029.pdf]

Supplementary Table 1. List of genes evaluated in the transcriptomic analysis.

Target	Forward primer	Reverse primer	Gene full name
ACTB	CCAACCGCGAGAAGATGAC	TAGCACAGCCTGGATAGCAA	Actin beta
AKT1	CACACACTCACCGAGAACC	TCGTGGGTCTGGAAAGAGTA	AKT serine/threonine kinase 1
BATF	AGCAGTGACTCCAGCTTCA	CTCTTCTGGGCGGCAATAC	Basic leucine zipper ATF-like transcription factor
BAX	GGGTTGTCGCCCTTTTCTAC	TCTTGGATCCAGCCCAACA	BCL2 associated X, apoptosis regulator
BCL2	ATGTGTGTGGAGAGCGTCAA	GTGCCGGTTCAGGTACTCA	BCL2 apoptosis regulator
BCL2L1	CTGCCTGCCTTTGCCTAA	CCAAAACACCTGCTCACTCA	BCL2 like 1
BCL6	GATGGAGCATGTTGTGGACAC	AGGAGGCTTGATGGCAGAAA	BCL6 transcription repressor
BECN1	GAGACCCAGGAGGAAGAGACTA	AATCTGCGAGAGACACCATCC	Beclin 1
CASP8	GGAAATCTCCAAATGCAAACTGG	CAGGATGACCCTCTTCTCCAT	Caspase 8
CCL3	ATGGCTCTCTGCAACCAGTT	CCGGGAGGTGTAGCTGAAG	C-C motif chemokine ligand 3
CCL3L1	ACGCAGGCAGCAAAGAGTA	ATTGGGAGCAGGTGATGGAA	C-C motif chemokine ligand 3 like 1
CCR5	TGAGACATCCGTTCCCCTACA	TGGCAGGGCTCCGATGTATA	C-C motif chemokine receptor 5
CCR7	GTGGTGGCTCTCCTTGTCA	CTGTGGTGTTGTCTCCGATGTA	C-C motif chemokine receptor 7
CD244	AACCACAGCCCTTCCTTCAA	GAGCAGGGTTCTGGGCTTTA	CD244 molecule
CD27	CACTACTGGGCTCAGGGAAA	TGCTGGTCACAGTCCTTCA	CD27 molecule
CD28	GTGGAGTCCTGGCTTGCTATA	GAGCCTGCTCCTCTTACTCC	CD28 molecule
CD38	ACCTCACATGGTGTGGTGAA	GTTGCTGCAGTCCTTTCTCC	CD38 molecule
CD69	TCACCCATGGAAGTGGTCAA	ACACACTTGTCAGACCCTGTA	CD69 molecule
CD8A	CCATCATGTACTTCAGCCACTTCG	GCTGCGACGCGATGGT	CD8a molecule
CDC42	GCTGTTGGTAAAACATGTCTCC	AACTGTGACTGCATAGTTGTCA	Cell division cycle 42
CDKN1b	GCAATGCGCAGGAATAAGGAA	TTGGGGAACCGTCTGAAACA	Cyclin dependent kinase inhibitor 1B
CPT1a	TCCATGCCATCCTGCTTTACA	AGTGGAATCGTGGATCCCAAA	Carnitine palmitoyltransferase 1A
CTLA4	CATGGACACGGGACTCTACA	AATCTGGGTTCCGTTGCCTA	Cytotoxic T-lymphocyte associated protein 4
CX3CR1	GTTGGTAGTGTTTGCCCTCAC	GGCCAGGTTCAGGAGGTAAA	C-X3-C motif chemokine receptor 1
CXCR5	ACGGAGACCTTCCTGTTCCA	ACGGCAAAGGGCAAGATGAA	C-X-C motif chemokine receptor 5
ENTPD1	AGGTGCCTATGGCTGGATTAC	GTCCAAAGCTCCAAAGGTTTCC	Ectonucleoside triphosphate diphosphohydrolase 1
EOMES	CTGTGGCAAAGCCGACAATA	CTCATCCAGTGGGAACCAGTA	Eomesodermin
ERCC1	AACCCGGGGCAAAATCCAA	CACATTGCGCACGAACTTCA	ERCC excision repair 1, endonuclease non-catalytic subunit
ERCC3	GACTGGAGTCCCTGATGGAA	TGCTTCAAGACCAGCTTGAC	ERCC excision repair 3, TFIIH core complex helicase subunit
ERCC4	CTTCTGGAATCTCTGAGAGCAA	GAGGTGCTGGAGTCAAGAAA	ERCC excision repair 4, endonuclease catalytic subunit
ESRRA	GGAGCGAGAGGAGTATGTTCTAC	CTTCTCGCAGCTGCTCCA	Estrogen related receptor alpha
FAS	GCATCTGGACCCTCCTACC	CCTTGGAGTTGATGTCAGTCAC	Fas cell surface death receptor
FASLG	TGGGGATGTTTCAGCTCTTCC	CTGTGTGCATCTGGCTGGTA	Fas ligand
FOXO1	GGTGTCAGGCTGAGGGTTA	TTCTCTCAGTTCCTGCTGTCA	Forkhead box O1
GAPDH	GAACGGGAAGCTTGTCATCAA	ATCGCCCCACTTGATTTTGG	Glyceraldehyde-3-phosphate dehydrogenase

GATA3	CACGGTGCAGAGGTACCC	AGGGTAGGGATCCATGAAGCA	GATA binding protein 3
GLS	AAGGCACAGACATGGTTGGTA	CACTGGCTGATTCACAAGTCAC	Glutaminase
GLS2	TTAGACCCACGGCGTGAA	TCGCCACTATAGGCAGCAA	Glutaminase 2
GZMB	CCCCATCCAGCCTATAATCCTAA	CTGGGCCTTGTTGCTAGGTA	Granzyme B
GZMK	CACATTTCATCTGGGCTTCTTAAA	GTGACACTTCTTTCCCTCCA	Granzyme K
HAVCR2	GGATCCAAATCCCAGGCATAA	CTTGGAAAGGCTGCAGTGAA	Hepatitis A virus cellular receptor 2
HIF1a	CAGTCGACACAGCCTGGATA	TTCTTCTGGCTCATATCCCATCAA	Hypoxia inducible factor 1 subunit alpha
HK1	TCATTTCCCTGCCAGCAGAC	CGCAGTCTGTTGCCTTAAAACC	Hexokinase 1
HLA-DRB1	AGAATGGAGACTGGACCTTCC	GCTCTGTGCAGATTCAGACC	Major histocompatibility complex, class II, DR beta 1
ID2	CTCAACACGGATATCAGCATCC	CACACAGTGCTTTGCTGTCA	Inhibitor of DNA binding 2
ID3	AAAAGGAGCTTTTGCCACTGAC	TTCCGGCAGGAGAGGTTCC	Inhibitor of DNA binding 3
IFITM1	ACACCCTCTTCTTGAACTGGT	CCAACCATCTTCCTGTCCCTA	Interferon induced transmembrane protein 1
IFNA1	TGACTCATACACCAGGTCAC	CAGGGGTGAGAGTCTTTGAA	Interferon alpha 1
IFNB1	ATGAGCAGTCTGCACCTGAA	GACTGTACTCCTTGGCCTTCA	Interferon beta 1
IFNg	ACTGCCAGGACCCATATGTAA	GTTCCATTATCCGCTACATCTGAA	Interferon gamma
IFNGR1	AAGCCAGGGTTGGACAAAA	GATATCCAGTTTAGGTGGTCCAA	Interferon gamma receptor 1
IL7R	GGAGAAAGTGGCTATGCTCAA	CTGCGATCCATTCACTTCCA	Interleukin 7 receptor
IRF1	AACAAGGATGCCTGTTTGTTCC	TGGGATCTGGCTCCTTTTCC	Interferon regulatory factor 1
KLRD1	AGCATTTACTCCAGGACCCAAC	TAACAGTTGCACCGGTACCC	Killer cell lectin like receptor D1
LAG3	TGGAGCCTTTGGCTTTCAC	GAGGGTGAATCCCTTGCTCTA	Lymphocyte activating 3
LAMP1	TCCAGGCTTTCAAGGTGGAA	CCACAGCGATGGGGATCA	Lysosomal associated membrane protein 1
LEF1	AAGAAAGTGCAGCTATCAACCA	GCTGTCTTTCTTTCCGTGCTA	Lymphoid enhancer binding factor 1
MIF	TGCCATCATGCCGATGTTCA	GGTGAGCTCGGAGAGGAAC	Macrophage migration inhibitory factor
MLST8	TGCAGGCTACGACCACAC	TGTGACCTCCAAGGCATTCA	MTOR associated protein, LST8 homolog
MTOR	CCAAACCCAGGTGTGATCAA	TCCTCATTTCCAGGCCACTA	Mechanistic target of rapamycin kinase
MX1	ATGCTACTGTGGCCCAGAAA	GGCGCACCTTCTCCTCATA	MX dynamin like GTPase 1
MYC	CCTGGTGCTCCATGAGGA	CCTGCCTCTTTTCCACAGAAA	MYC proto-oncogene, bHLH transcription factor
NDUFA5	ACCAACTTCAAGGCGGTCAA	TGGCTCCCATAGTTTCCATTCC	NADH:ubiquinone oxidoreductase subunit A5
NFATC1	TCCTCTCCAACACCAAAGTCC	AGGATTCCGGCACAGTCAA	Nuclear factor of activated T cells 1
NT5E	ATGAACGCCCTGCGCTAC	GTGGCTCGATCAGTCCTTCC	5'-nucleotidase ecto
OAS1	TACCCTGTGTGTGTGTCCAA	AGAGGACTGAGGAAGACAACC	2'-5'-oligoadenylate synthetase 1
OPA1	CCAAAGTAGACCTGGCAGAGAA	TCATTGGGAAGAGCTTTCCTTCA	OPA1, mitochondrial dynamin like GTPase
PCNA	TCTGAGGGCTTCGACACCTA	CATTGCCGGCGCATTTTAGTA	Proliferating cell nuclear antigen
PDCD1	GCAGCCTGGTGCTGCTA	GTGCGCCTGGCTCCTA	Programmed cell death 1
PFKM	AGGAAGAATGTGCTTGGTCAC	CGCCCATCTTAGTGGCAAA	Phosphofructokinase, muscle
PKM	CGGGTGAACTTTGCCATGAA	CATCCGGTCAGCACAATGAC	Pyruvate kinase M1/2
POLR2A	CTCGCCTCTTCTACTCCAACA	ATGGAGTCCCCAATGCCAATA	RNA polymerase II subunit A
PRDM1	CCTGGTACACACGGGAGAAAA	TTGAGATTGCTGGTGCTGCTA	PR/SET domain 1
PRF1	CATCTGTGTAGCCGCTTCTCTA	TGCCCAGGAGGAGCAGAC	Perforin 1

PRKAA1	CCAACTATGCTGCACCAGAA	AGAATAACCCCACTGCTCCA	Protein kinase AMP-activated catalytic subunit alpha 1
PRKCA	ACCATCCGCTCCACACTAAA	AGTCGTCGGTCTTTGTCTGAA	Protein kinase C alpha
RHOA	GTGCCCACAGTGTTTGAGAA	TGTGTCCCACAAAGCCAAC	Ras homolog family member A
RICTOR	CTTCCGTGTCGGAGGTTCATA	ACACAGCCTCTGCTTCTTCA	RPTOR independent companion of MTOR complex 2
RPTOR	GCTCAGAGCTGGAGGATGAA	AGGGTCCACACCAACATTCA	Regulatory associated protein of MTOR complex 1
RUNX1	ACCACAGAGCCATCAAAATCAC	CGGGCTTGGTCTGATCATCTA	RUNX family transcription factor 1
SH2D1A	GAAGTCCTCAGCTAGAAGTACACA	GGGCTTTCAGGCAGACATCA	SH2 domain containing 1A
SLC2A1	ATTGTGGGCATGTGCTTCC	AGAACCAGGAGCACAGTGAA	Solute carrier family 2 member 1
SLC2A3	CCGTCGGACTCTTCGTCAA	TAAAGCAGCCACCAGTGACA	Solute carrier family 2 member 3
STAT3	GGAAATAATGGTGAAGGTGCTGAAC	CCGAGGTCAACTCCATGTCAAA	Signal transducer and activator of transcription 3
TBX21	GGGCGTCCAACAATGTGAC	CCGTCGTTCACCTCAACGATA	T-box transcription factor 21
TCF7	CTGCACATGCAGCTATACCC	TGGATTCTTGGTGCTTTTCCC	Transcription factor 7
TFAM	GAAGACTGTAAAGGAAAACTGGAA	CATTTGTTCTTCCCAAGACTTCA	Transcription factor A, mitochondrial
TIGIT	GTGGTGGTCGCGTTGACTA	TCCTGTCCAGCTGATTTTCTCC	T cell immunoreceptor with Ig and ITIM domains
TNF	CCCAGGGACCTCTCTCTAATCA	ATGGGCTACAGGCTTGTCAC	Tumor necrosis factor
TNFSF10	AGAAGGAAGGGCTTCAGTGAC	CCTGGACCTCCATCATAGCC	TNF superfamily member 10
TOX	CTGAAGCAACTCGCAGCATA	GTCTTCACGTCAACAGGTTCA	Thymocyte selection associated high mobility group box
TP53	GACTGTACCACCATCCACTACA	AAAGCTGTTCCGTCCCAGTA	Tumor protein p53
VHL	TGTTTAGGGGCAAACATCACAA	AAAATGCCACCACCTTCTCC	Von Hippel-Lindau tumor suppressor
XCL1	ACTGCCGGTTAGCAGAATCAA	CCCATGTGGCTTGTGGATCA	X-C motif chemokine ligand 1
XPC	CTCTGCTCGAGATGATGAGGAA	ACAATACCAGCCGGGTCAA	XPC complex subunit, DNA damage recognition and repair factor
ZEB2	AGGCCAATGGGCAAGAAGAA	AGGTCAGCAGTTGGGCAAA	Zinc finger E-box binding homeobox 2

Supplementary Table 2. Comparison of gene expression in reprogrammed vs non-reprogrammed memory CD8^{+} T cells

Cell subset	TCM		TTM		TEM		TTE	
Condition	Unst.	Anti-CD3/CD28	Unst.	Anti-CD3/CD28	Unst.	Anti-CD3/CD28	Unst.	Anti-CD3/CD28
ACTB	0.39314	0.98717	**0.00950**	0.31392	0.24186	0.29836	**0.02025**	0.38121
BECN1	0.78280	0.55642	0.05596	0.73645	0.77796	0.07879	0.20553	0.48742
CD27	0.44104	0.89127	0.61684	0.93423	0.26404	0.22998	0.93395	0.54778
HAVCR2	**0.00997**	**0.03159**	0.59108	0.11892	0.15595	0.60136	0.77540	0.58720
ERCC3	0.12331	0.76597	0.43887	0.51193	0.93794	0.38003	0.09848	0.27662
GLS	**0.04843**	0.92576	0.82708	0.45366	0.39786	0.31850	0.73614	0.70576
IFITM1	0.43695	0.05963	0.16824	0.09358	**0.00831**	0.48175	0.42403	0.14627
LAG3	0.09012	0.40100	**0.03511**	0.93125	**0.03941**	0.14642	0.80453	**0.00426**
NDUFA5	0.55774	0.63930	0.06901	0.15976	0.72687	0.32244	0.05617	0.35681
PKM	0.48923	0.57563	0.37442	0.67914	0.07508	0.95257	**0.00259**	0.84342
RPTOR	0.88194	0.62363	0.23248	0.68207	0.08728	0.73072	**0.01672**	0.85393
ZEB2	0.52301	0.52652	0.53700	0.73928	**0.00852**	0.26775	0.12723	0.99675
AKT1	0.92849	0.88008	0.38864	0.31517	0.62700	0.78491	0.11944	0.95594
CASP8	0.98212	0.63577	0.83182	0.10928	0.84958	0.90898	0.61627	0.38533
CXCR5	0.37876	0.31548	0.06508	0.14407	0.17804	0.22674	0.28741	0.06544
ID3	0.15024	0.70751	0.76671	0.31276	0.36117	0.11142	0.05317	**0.03198**
ERCC4	0.91570	0.31063	0.19540	0.29352	0.65727	**0.00953**	0.59048	**0.00699**
GLS2	**0.04139**	0.26316	0.89919	0.19937	**0.01575**	0.70460	**0.00669**	**0.01796**
IFNA1	0.11437	0.35875	**0.01855**	**0.00023**	0.74326	0.06711	0.77279	0.06557
LAMP1	0.44974	0.54170	0.61946	0.63230	0.70472	0.70756	0.66329	0.82375
NFATC1	0.37160	0.73934	0.10285	0.14353	0.56749	0.86671	0.48270	0.79177
POLR2A	0.36077	0.41262	0.27286	0.24257	**0.01714**	0.83868	**0.00269**	0.25789
RUNX1	0.16363	0.84356	0.15572	0.57026	0.92926	**0.01218**	0.16985	0.10652
TFAM	0.50097	0.22916	0.08383	0.14640	0.49536	0.68245	0.69893	0.31635
CD28	0.59164	0.70760	0.44380	0.41748	0.73450	0.98896	0.91278	0.37350
CCL3	**0.03660**	0.63192	0.58924	0.70402	0.50691	0.96454	0.28758	0.77203
CD38	0.39081	0.62312	0.14847	0.99527	0.50564	0.29327	**0.03638**	**0.01607**
TIGIT	0.44893	0.08024	0.05029	0.16029	**0.04147**	**0.03815**	0.85979	**0.00058**
ESRRA	**0.00074**	0.34222	0.64415	0.83620	0.12089	0.33939	0.67974	0.92574
GZMB	0.42031	**0.02960**	0.80365	0.28852	0.46204	0.51374	0.05837	0.74284
IFNB1	0.27765	0.72803	0.21161	0.16718	0.11377	0.69546	0.52235	0.14556
LEF1	0.25218	0.37727	0.92834	0.80648	0.97620	0.36986	**0.02389**	0.05101
NT5E	0.91590	0.76453	0.82247	0.62947	0.78001	0.95188	0.22263	0.36665
PRDM1	0.27766	0.09738	0.11201	0.06875	0.90200	0.92176	0.52394	0.60923
SH2D1A	0.97686	0.80239	0.05178	0.88396	0.87157	0.07729	0.22502	0.76657
TNF	0.13315	0.27229	0.24586	0.62660	0.86016	0.67062	0.27525	0.20224
BATF	**0.01057**	0.59668	0.91498	0.13129	0.68683	**0.03797**	0.03212	0.09602
CCL3L1	0.35535	0.24487	0.75912	0.63275	0.18949	0.49881	0.78161	0.94770
CD69	0.36426	0.61473	0.77202	0.89000	0.15002	0.26970	0.91514	0.12058
CDKN1b	0.14062	0.88667	0.15171	0.28399	0.09685	0.66130	0.97064	0.07270
FAS	0.24415	0.59937	**0.02220**	0.08000	0.07447	0.99614	0.79199	0.06500
GZMK	**0.00196**	0.86111	0.78385	0.49315	0.79655	0.58445	0.33569	0.98948
IFNG	**0.03201**	0.85804	0.23627	0.37687	0.13751	0.89184	0.29837	0.48762
MIF	0.88814	0.97356	**0.03975**	**0.00062**	0.98307	**0.01120**	0.43865	0.42672
OAS1	**0.02163**	0.91486	0.91040	0.25828	0.20229	0.84763	0.06447	0.05337
PRF1	0.88757	0.21382	0.88858	0.60435	0.27232	0.73094	0.21211	0.77064
SLC2A1	0.18798	0.49723	**0.01567**	0.87762	0.83211	0.25502	0.26956	0.68709

TNFSF10	0.94041	0.32709	0.82157	0.42123	0.85318	0.22215	0.76597	**0.02479**
BAX	**0.02494**	0.19604	0.87124	0.89468	0.54797	0.06819	0.28652	0.36608
CX3CR1	**0.02498**	0.11974	0.13380	**0.00028**	**0.04985**	0.66422	0.39722	0.61718
CD8A	**0.02779**	0.61961	0.61890	0.42438	0.72466	0.62932	0.44703	0.44629
TOX	0.67495	0.20496	0.46805	0.65960	0.67387	0.20329	0.50108	0.35948
FASLG	0.48155	**0.04982**	**0.00406**	0.79784	0.21781	0.87808	**0.00018**	0.06595
HIF1A	0.60429	0.41168	0.98560	0.46804	0.55077	0.41247	0.30757	0.24452
IFNGR1	**0.04319**	0.16194	0.41767	0.12793	0.72174	0.55399	0.44818	0.30488
MLST8	**0.02882**	0.25165	**0.03102**	0.34927	0.15959	0.76705	0.46550	0.46487
OPA1	**0.03522**	0.79821	0.20740	0.49980	0.06194	0.67251	0.09880	0.94127
PRKAA1	**0.00214**	**0.03177**	0.37779	0.50090	0.67168	0.19595	0.07021	0.64607
SLC2A3	0.89209	0.38396	**0.00632**	0.68719	0.55114	0.60494	**0.03613**	0.89941
TP53	**0.00206**	0.57318	0.68530	0.97694	0.11830	0.57670	0.11814	0.80863
BCL2	0.35122	**0.03261**	0.06022	**0.00829**	0.83238	**0.02248**	0.63514	0.66415
CDC42	0.96920	0.18449	0.92388	0.01260	0.96956	**0.01834**	0.53706	0.18247
CPT1a	0.89059	0.80586	0.12233	0.85818	0.27724	0.86680	**0.00376**	0.65464
FOXO1	0.44484	0.48357	**0.02708**	0.82179	0.98764	0.64759	0.80284	0.20942
HK1	**0.02060**	0.54612	0.35068	0.47818	0.96827	**0.04198**	0.15982	0.08508
IL7R	0.88963	0.43114	0.27002	0.95649	0.74299	0.05326	0.09987	0.42098
mTOR	0.47627	0.72088	0.89413	0.40504	0.90665	0.89978	**0.01174**	0.68539
PCNA	0.13072	0.10048	0.09623	0.65842	0.86322	0.38040	0.97902	0.85349
PRKCA	0.95157	0.05074	**0.02886**	0.41887	0.26435	0.63399	**0.02300**	**0.00747**
STAT3	**0.03224**	0.70897	**0.04400**	0.96048	0.38415	0.53476	0.25269	0.66773
VHL	0.95018	0.18788	0.68506	0.28807	0.11726	0.19256	0.94120	**0.01543**
BCL2L1	**0.00313**	0.36768	0.55142	0.64430	0.64439	0.30852	0.08739	0.33306
CCR7	0.94868	0.61199	0.33266	0.32997	0.43064	0.29261	0.61666	0.66241
CTLA4	0.88680	0.37995	0.90181	**0.00027**	0.23599	0.06855	0.75055	**0.02575**
IRF1	0.12329	0.62707	0.27072	0.31171	0.15329	0.47067	0.78863	0.42417
PDCD1	0.13271	0.06656	0.81805	0.31982	0.89619	0.56785	0.47398	0.54510
RHOA	0.17382	0.91145	0.97654	0.21839	0.22438	0.65182	0.55127	0.50175
TBX21	**0.03166**	0.21296	0.65616	0.65371	0.59122	0.05550	0.81579	0.30459
XCL1	**0.01214**	0.07730	0.16446	0.78185	0.71803	0.19051	0.87346	0.38354
BCL6	0.09734	0.15765	0.74798	0.40291	0.06656	0.77596	0.71865	0.89754
CD244	**0.01214**	0.07730	0.49663	0.49177	0.18478	0.68653	0.26929	0.74896
EOMES	**0.02920**	0.17397	0.08447	0.47522	0.29636	0.91581	0.18110	0.50142
ERCC1	0.16055	0.88544	0.44704	0.93166	**0.01177**	0.23556	0.65480	0.88074
GATA3	0.25522	0.26545	0.33243	0.55130	0.46216	0.06514	0.38486	0.63015
ID2	0.41570	0.20982	0.83045	**0.00541**	0.40547	0.24681	0.23141	0.06895
KLRD1	0.24483	0.92396	0.94743	0.36775	0.07814	0.12302	0.94568	0.81870
RICTOR	0.93610	0.16491	**0.02842**	0.07998	0.17505	0.12363	0.94691	0.82140
TCF7	0.05479	0.51363	0.91555	0.92147	0.90917	0.39660	0.14663	0.56725
XPC	0.84713	0.31596	**0.00072**	**0.03588**	0.20746	0.06412	0.35559	0.59213

P values obtained after comparison of gene expression of sorted memory $CD8^+$ T cells treated with DMSO vehicle control versus GSK3 inhibitor, followed by resting (Unst.) or stimulation with anti-CD3/CD28 for 48 hs.

Supplementary Table 3. Clinical characteristics of people with HIV included in the study

ID	Age at inclusion (years)	Gender	Nadir $CD4^+$ T cell count pre-ART (cells/μL)	Viral load pre-ART (RNA copies/μL)	$CD4^+$ T cell count at inclusion (cells/μL)	Viral load at inclusion (RNA copies/μL)	ART duration at inclusion (years)
mTOR_34001TT	56	Male	Not available	Not available	962	<40	8
mTOR_34002TT	57	Male	180	31 352	678	<40	22
mTOR_34003TT	61	Male	243	2 239 000	919	<40	7.5
mTOR_34008TT	47	Female	191	54 230	727	<40	18
mTOR_44001TT	56	Male	513	20 038	1,030	<40	12.5
mTOR_44002TT	55	Male	16	285 000	415	<40	3
mTOR_44004TT	54	Male	203	10 000	525	<40	5.5
mTOR_44005TT	51	Female	65	220 000	957	<40	19
mTOR_44007TT	54	Female	32	59 426	678	<40	19
mTOR_44008TT	50	Male	3	349 000	592	<20	9
mTOR_44009TT	64	Male	608	6 320	942	<20	2
mTOR_44010TT	45	Male	264	500 000	791	<20	11
mTOR_44011T	59	Male	80	628 000	634	<40	15
mTOR_54002TT	54	Male	6	400 000	390	<20	2
mTOR_54004TT	72	Male	641	2 505 600	1,032	<20	8
mTOR_54007TT	57	Male	292	383 834	501	<20	2
9401/11	42	Female	563	40 738	1,282	<20	19
7508/03	29	Male	550	3 981	550	<20	23
7513/09	37	Male	371	28 183	519	<20	19
7507/04	36	Male	708	10 000	989	<20	23

Supplementary Table 4. List of flow cytometry antibodies

Target	Fluorophore	Company	Clone	Cat number	Dilution 1/
For phenotyping					
CD3	APC efluor 780	Thermo Fisher	UCHT1	47-0038-42	50
CD3	Alexa Fluor 700	BD	UCHT1	557943	50
CD8	PE Texas red	Thermo Fisher	3B5	MHCD0817	100
CD8	BUV496	BD	RPA-T8	612942	200
CD4	BUV737	BD	OKT4	750977	200
CCR7	PE Cy7	BD	3D12	557648	40
CD27	PerCP Cy5.5	BD	M-T271	560612	40
CD27	PE	BD	M-T271	560985	40
CD45RA	APC H7	BD	HI100	560674	80
CD28	BV711	BD	CD28.2	563131	80
CD95	APC	BD	DX2	561978	40
CD127	Alexa Fluor 488	Biolegend	A019D5	351314	80
HLA-DR	Superbright 780	Thermo Fisher	LN3	78995642	40
CD38	Superbright 600	Thermo Fisher	HB7	63038842	40
TIM-3	PE Cy7	Thermo Fisher	F38-2E2	25-3109-42	40
LAG-3	APC efluor 780	Thermo Fisher	3DS223H	47-2239-42	40
LAG-3	BUV395	BD	T47-540	745640	80
TIGIT	BV786	BD	741182	747838	40
PD-1	BV421	BD	EH12.1	562516	80
PD-1	BUV661	BD	EH12.1	750260	80
CD215 (IL-15R alpha)	FITC	Thermo Fisher	eBioJM7A4	11-7159-42	80
CD122 (IL-15R beta)	PE	BD	Mik-β2	557323	80
Transcription factors					
TCF-1	PE	BD	O4-46	564217	20
T-bet	V450	BD	S33-966	561312	80
TOX	eFluor 660	Thermo Fisher	TXRX10	50-6502-82	80
BLIMP1	CF594	BD	6D3	565274	20
BCL6	Alexa Fluor 488	BD	K112-91	561524	20
Eomesodermin	APC	R&D	644730	IC6166A	40
Beta-catenin	Alexa Fluor 488	Thermo Fisher	15B8	53-2567-41	20
Effector function					
TNF-α	PerCP Cy5.5	BD	MAb11	560679	40
Granzyme B	Alexa Fluor 647	BD	GB11	560212	80
IL-2	APC-R700	BD	MQ1-17H12	565136	40
IFN-γ	PE Cy7	BD	4S.B3	557844	200
IFN-γ	V450	BD	B27	560371	80
IFN-γ	BV605	BD	B27	562974	40
CD107a	FITC	BD	H4A3	555800	40
CD107a	BV786	BD	H4A3	563869	200
Phospho proteins					
phospho AKT Ser 473	Alexa Fluor 488	Cell signaling	D9E	4071S	50
phospho S6 S235/236	Pacific blue	Cell signaling	D57.2.2E	8520S	50
For sorting					
CD3	Alexa Fluor 700	BD	UCHT1	557943	50
CD8	APC Cy7	BD	RPA-T8	557760	50
CCR7	PE Cy7	BD	3D12	557648	40
CD27	PE	BD	M-T271	560985	40
CD45RA	BV421	BD	HI100	562885	40

Supplementary figure legends

Supplementary Figure 1. A. Flow cytometry gating strategy used to identify CD8$^+$ T cell subpopulations: Naïve, Stem cell memory (TSCM), Central memory (TCM), Transitional memory (TTM), Effector memory (TEM), and Terminal effector (TTE). **B.** CD8$^+$ T cells from people without HIV were cultured with the GSK3 inhibitor BIO at increasing concentrations (0 to 5 μM; all for 12 hs) or incubation times (0 to 72 hs; all at 3 μM), without further stimulation, to assess the effect on cell death and on the expression of CCR7 and TCF-1. Treatment at 3 μM for 12 hs was chosen since, under these conditions, the cells exhibited the highest upregulation of CCR7 and TCF-1, without toxicity. **C.** Expression of CCR7, CD27 and TCF-1 in total CD8$^+$ T cells after treatment with vehicle control, or the GSK3 inhibitors BIO or TWS119, for 12 hs. The median fluorescence intensity of each marker is depicted. **D.** Fold change in TSCM, TCM and TEM cells induced by BIO or TWS119, relative to vehicle control (n=3 people without HIV). **E.** Fold change in T-bet$^+$ cells among TSCM and TCM induced by anti-CD3/CD28 stimulation, relative to vehicle control, in cells previously treated with vehicle control, BIO or TWS119 (n=3). **F.** Fold change in T-bet$^+$ cells among naïve/TSCM induced by anti-CD3/CD28 stimulation (0.2 and 1 μg/mL), relative to unstimulated cells, previous treatment with vehicle control or the GSK3 inhibitor (n=3). **G.** Fold change in T-bet$^+$ cells among each CD8$^+$ T cell subpopulation induced by anti-CD3/CD28 + ICAM-1 stimulation, relative to unstimulated cells (n=5; Wilcoxon test). **H.** Expression of granzyme B, IL-2, IFN-γ and TNF-α in CD8$^+$ T cells responding to anti-CD3/CD28 stimulation for 48 hs by the expression of the indicated effector molecules (n=5; data from three independent experiments). **I.** Frequency of IL-2$^+$ TNF-α^+ cells among each CD8$^+$ T cell subpopulation after anti-CD3/CD28 + ICAM-1 stimulation (n=5; Wilcoxon test). **J.** After reprogramming with the GSK3 inhibitor, CD8$^+$ T cells were stained with CFSE, and then stimulated for 48 hs with anti-

CD3/CD28, followed by wash and resting for 5 days. Then, cells were restimulated for another 48 hs with anti-CD3/CD28. Left: Analysis of proliferation (CFSE dilution). Right: Frequencies of $CD8^+$ T cells with 0, 1-3 or ≥4 cell divisions at the end of the re-stimulation (n=9; Wilcoxon test; data from two independent experiments). **K.** Frequency of $PD\text{-}1^+$ $LAG\text{-}3^+$ $TIM3^+$ $TIGIT^+$ cells in total $CD8^+$ T cells after restimulation (n=6; data from two independent experiments). **L**. After reprogramming with the GSK3 inhibitor, $CD8^+$ T cells were left unstimulated, stimulated once for 48 hs with anti-CD3/CD28, or stimulated twice with anti-CD3/CD28. The expression of T-bet in TCM and TTM cells is shown (n=5; Wilcoxon test; data from two independent experiments). **P<0.05*. ***P≤0.01*; *NS:* Not statistically significant.

Supplementary Figure 2. Heat maps of genes differentially expressed in reprogrammed versus non-reprogrammed TTM (**A**), TEM (**B**), and TTE (**C**) cells. The scale on the heatmap indicates the mRNA levels relative to the housekeeping gene (*GAPDH*) and plotted as log $2^{-\Delta Ct}$, in unstimulated and polyclonally-stimulated cells. At least 6 people without HIV were included for each comparison; data from two independent experiments.

Supplementary Figure 3. A. After treatment with the GSK3 inhibitor, cells from people with HIV (n=12) were stimulated for 6 hs with Gag peptides, for the analysis of $TNF\text{-}\alpha^+$ HIV-specific $CD8^+$ T cells (n=12; ****P≤0.001,* Wilcoxon test; data from three independent experiments). **B.** Representative flow cytometry analysis (from a total of 6 donors) of the viability of proliferating HIV Gag-specific $CD8^+$ T cells, after stimulation for 5.5 days with Gag peptides, and re-stimulated with the same peptides for another 12 hs.

Supplementary Figure 4.

A-B. After treatment with the GSK3 inhibitor, cells from people without HIV (n=6, data from three independent experiments) were stimulated for 6 hs (**A**) or 6 days (**B**) with HCMV pp65 peptides, for the analysis of the total frequency (IFN-γ^+ or CD107a$^+$ or TNF-α^+) of antigen-specific CD8$^+$ T cells. **C-D.** Proportion of memory subpopulations (**C**), and the expression of IFN-γ, CD107a, and TNF-α on a per cell basis (**D**) in HCMV pp65-specific CD8$^+$ T cells, after 6 hs peptide stimulation. **E-F.** Cells from people with HIV (n=4) were stimulated for 6 hs with HCMV pp65 or HIV Gag peptides, for the analysis of CD107a$^+$, IFN-γ^+ or TNF-α^+ antigen-specific CD8$^+$ T cells. In **F**, the Δ change in TNF-α production between reprogrammed / non-reprogrammed HCMV and HIV-specific cells is shown. *$P<0.05$; **$P\leq0.01$; *NS:* Not statistically significant; Wilcoxon test.

Supplementary Figure 5. A. Flow cytometry analysis of the expression of pS6 in CD8$^+$ T cells from a person with HIV (representative from a total of 7 donors), in the absence of stimulation, or stimulated with Gag peptides for 6 hs. The % of pS6$^+$ cells in total and IFN-γ^+ CD8$^+$ T cells, as well as the median fluorescence intensity (MFI) of pS6 in pS6$^+$ cells is shown. **B.** Left: Flow cytometry analysis of the expression of CD107a and IFN-γ after vehicle control or GSK3 inhibitor treatment, followed by Gag peptides stimulation for 6 hs in the presence or absence of glucose. Right: Frequency of the total HIV Gag-specific CD8$^+$ T cell response (IFN-γ^+ or CD107a$^+$ or IL-2$^+$ or TNF-α^+) (n=6). **C.** Ratio of the frequency of the total HIV Gag-specific CD8$^+$ T cell response between no glucose versus glucose conditions. **D.** Frequency of TNF-α^+ HIV Gag-specific CD8$^+$ T cells after Gag peptides stimulation for 6 hs in the absence of glucose (n=6). *$P<0.05$; *NS:* Not statistically significant; Wilcoxon test. Data from two independent experiments.

Supplementary Figure 6. A. HIV suppressive capacity of non-reprogrammed and reprogrammed $CD8^+$ T cells (Log10 decrease of $KC57^+$ $CD4^+$ T cells). **B.** Representative flow cytometry analysis (from a total of 4 donors) of IFN-γ^+ HIV-specific $CD8^+$ T cells evaluated after 7 days of coculture with HIV-1 superinfected $CD4^+$ T cells. *$P<0.05$; Wilcoxon test.

Supplementary Figure 7. Expression of CD122 (**A**) and CD215 (**B**) in each $CD8^+$ T cell subpopulation (n=5 people without HIV, data from two independent experiments). **C**. Fold change in the expression of T-bet in response to IL-15 (relative to unstimulated cells) in HIV $dextramer^+$ $CD8^+$ T cells from non-controllers (n=4; data from three independent experiments). *$P<0.05$; *NS:* Not statistically significant; Wilcoxon test.

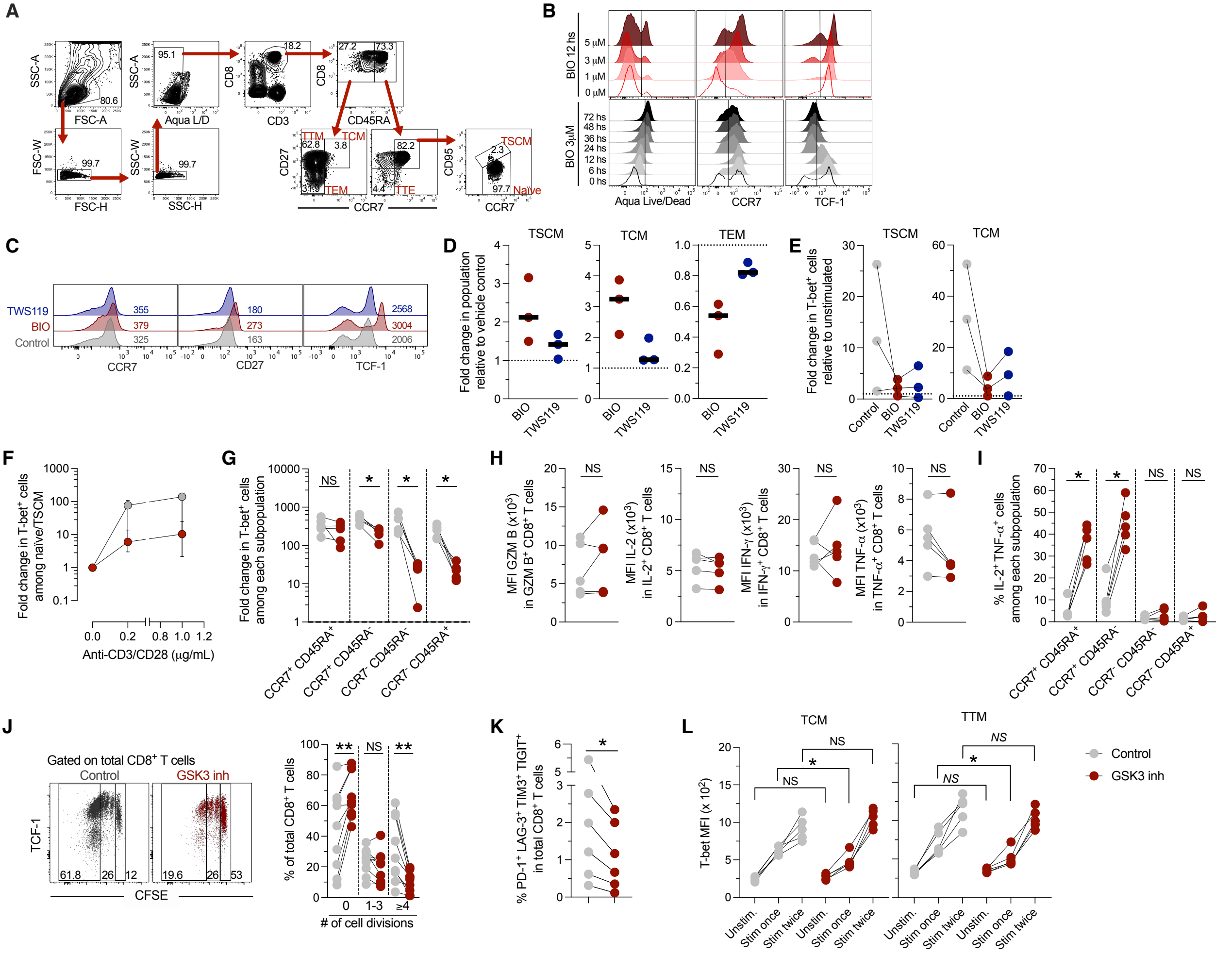
A
SSC-A
FSC-A
80.6
FSC-W
FSC-H
99.7
Aqua L/D
95.1
SSC-W
SSC-H
99.7
CD8
CD3
18.2
27.2
73.3
CD45RA
CD27
TTM
TCM
62.8
3.8
31.9
TEM
82.2
4.4
TTE
CCR7
CD95
TSCM
2.3
97.7
Naïve
B
BIO 12 hs
5 µM
3 µM
1 µM
0 µM
BIO 3µM
72 hs
48 hs
36 hs
24 hs
12 hs
6 hs
0 hs
Aqua Live/Dead
CCR7
TCF-1
C
TWS119
BIO
Control
355
379
325
180
273
163
2568
3004
2006
CCR7
CD27
TCF-1
D
Fold change in population relative to vehicle control
TSCM
TCM
TEM
BIO
TWS119
E
Fold change in T-bet+ cells relative to unstimulated
TSCM
TCM
Control
BIO
TWS119
F
Fold change in T-bet+ cells among naïve/TSCM
Anti-CD3/CD28 (µg/mL)
G
Fold change in T-bet+ cells among each subpopulation
NS
*
*
*
CCR7+ CD45RA+
CCR7+ CD45RA-
CCR7- CD45RA-
CCR7- CD45RA+
H
MFI GZM B (x10³) in GZM B+ CD8+ T cells
MFI IL-2 (x10³) in IL-2+ CD8+ T cells
MFI IFN-γ (x10³) in IFN-γ+ CD8+ T cells
MFI TNF-α (x10³) in TNF-α+ CD8+ T cells
NS
NS
NS
NS
I
% IL-2+ TNF-α+ cells among each subpopulation
*
*
NS
NS
CCR7+ CD45RA+
CCR7+ CD45RA-
CCR7- CD45RA-
CCR7- CD45RA+
J
Gated on total CD8+ T cells
Control
GSK3 inh
TCF-1
CFSE
61.8
26
12
19.6
26
53
% of total CD8+ T cells
**
NS
**
0
1-3
≥4
# of cell divisions
K
% PD-1+ LAG-3+ TIM3+ TIGIT+ in total CD8+ T cells
*
L
TCM
TTM
T-bet MFI (x 10²)
NS
*
NS
Unstim.
Stim once
Stim twice
Control
GSK3 inh

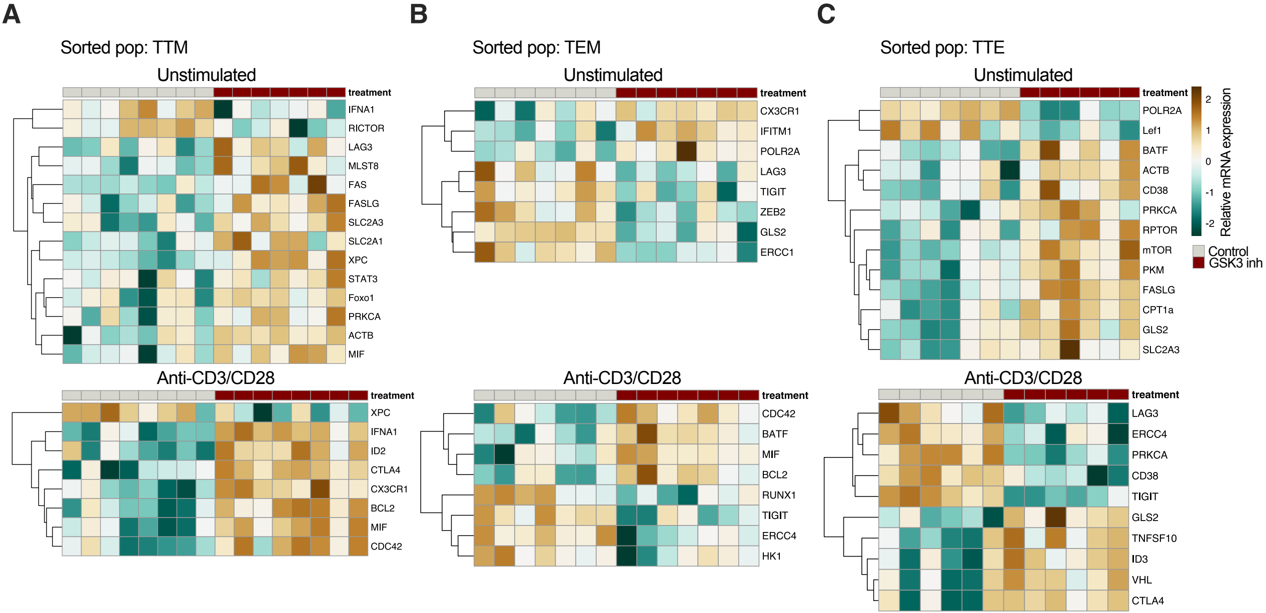

A
Sorted pop: TTM
Unstimulated
treatment
IFNA1
RICTOR
LAG3
MLST8
FAS
FASLG
SLC2A3
SLC2A1
XPC
STAT3
Foxo1
PRKCA
ACTB
MIF
Anti-CD3/CD28
treatment
XPC
IFNA1
ID2
CTLA4
CX3CR1
BCL2
MIF
CDC42
B
Sorted pop: TEM
Unstimulated
treatment
CX3CR1
IFITM1
POLR2A
LAG3
TIGIT
ZEB2
GLS2
ERCC1
Anti-CD3/CD28
treatment
CDC42
BATF
MIF
BCL2
RUNX1
TIGIT
ERCC4
HK1
C
Sorted pop: TTE
Unstimulated
treatment
POLR2A
Lef1
BATF
ACTB
CD38
PRKCA
RPTOR
mTOR
PKM
FASLG
CPT1a
GLS2
SLC2A3
Anti-CD3/CD28
treatment
LAG3
ERCC4
PRKCA
CD38
TIGIT
GLS2
TNFSF10
ID3
VHL
CTLA4
2
1
0
-1
-2
Relative mRNA expression
Control
GSK3 inh

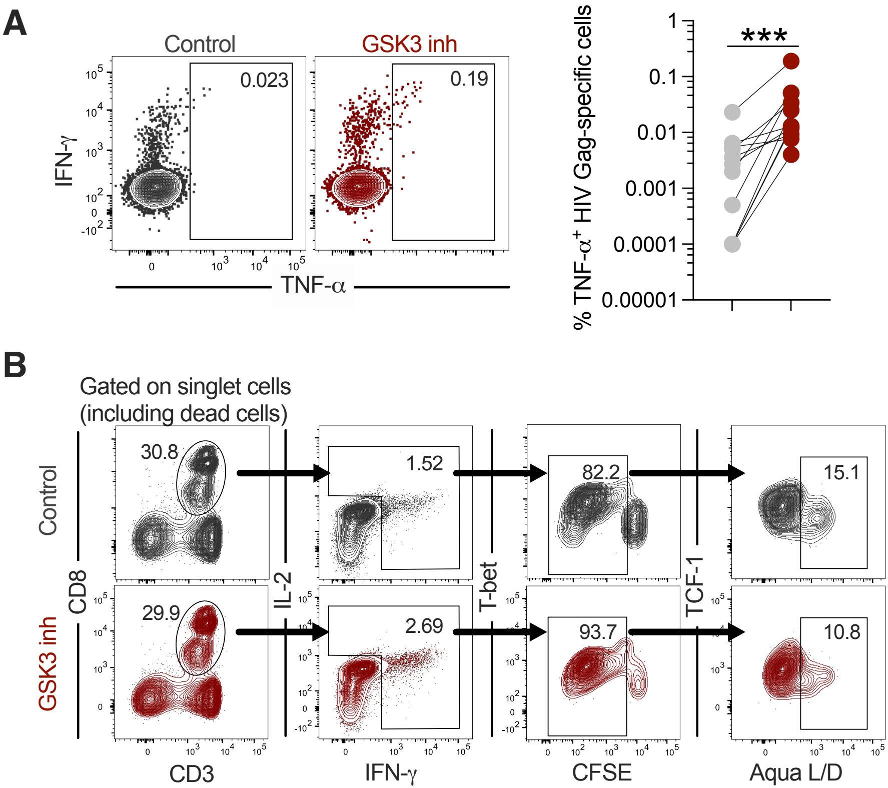

A
Control
GSK3 inh
0.023
0.19
IFN-γ
TNF-α
% TNF-α+ HIV Gag-specific cells
***
1
0.1
0.01
0.001
0.0001
0.00001
B
Gated on singlet cells
(including dead cells)
Control
GSK3 inh
CD8
CD3
30.8
29.9
IL-2
IFN-γ
1.52
2.69
T-bet
CFSE
82.2
93.7
TCF-1
Aqua L/D
15.1
10.8

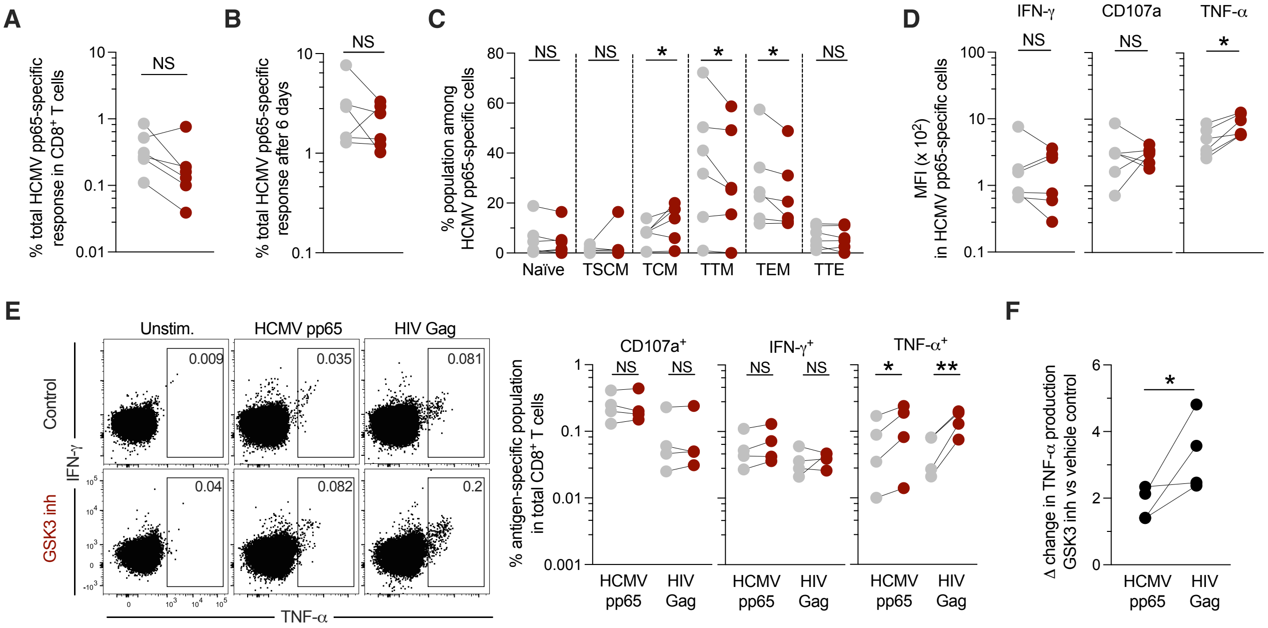

A
% total HCMV pp65-specific response in CD8+ T cells
NS
B
% total HCMV pp65-specific response after 6 days
NS
C
% population among HCMV pp65-specific cells
NS
*
Naïve
TSCM
TCM
TTM
TEM
TTE
D
MFI (x 10²) in HCMV pp65-specific cells
IFN-γ
CD107a
TNF-α
E
Unstim.
HCMV pp65
HIV Gag
Control
GSK3 inh
IFN-γ
TNF-α
0.009
0.035
0.081
0.04
0.082
0.2
% antigen-specific population in total CD8+ T cells
CD107a+
IFN-γ+
TNF-α+
**
HCMV pp65
HIV Gag
F
Δ change in TNF-α production GSK3 inh vs vehicle control

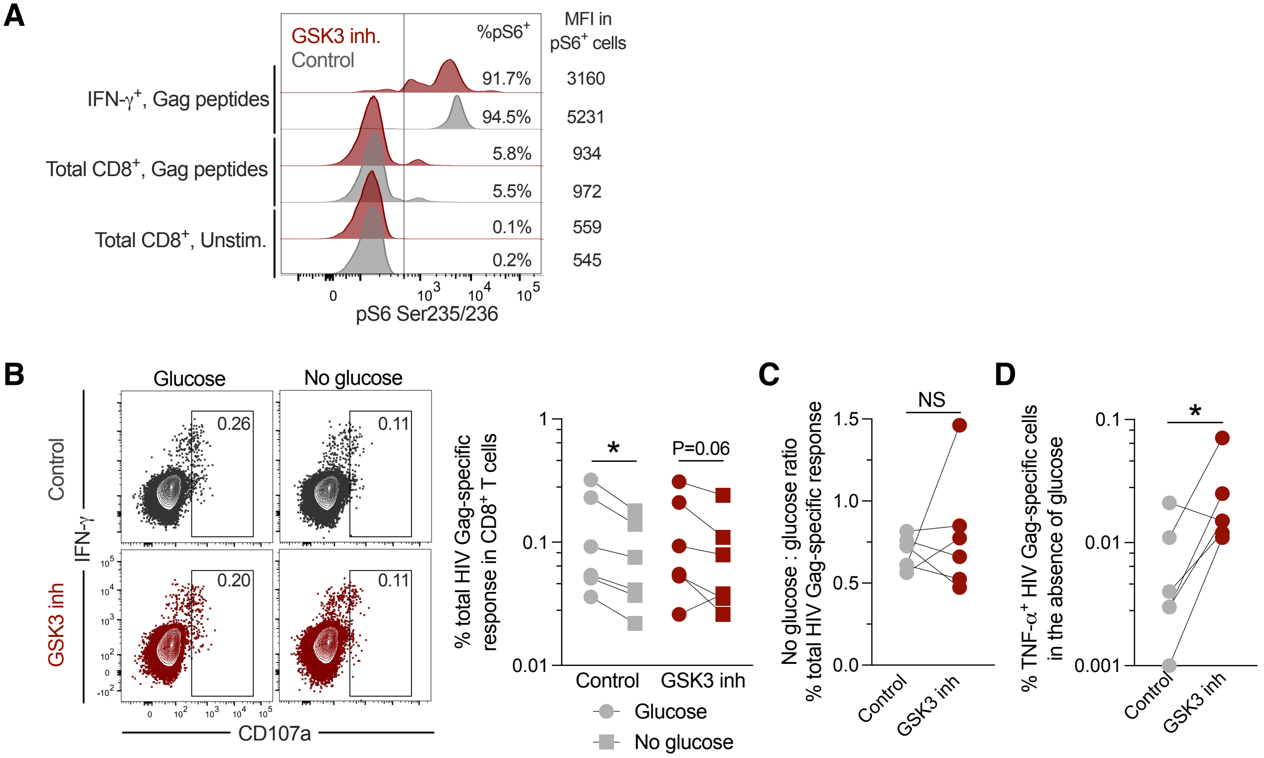

A
GSK3 inh.
Control
%pS6+
MFI in pS6+ cells
IFN-γ+, Gag peptides
Total CD8+, Gag peptides
Total CD8+, Unstim.
91.7% 3160
94.5% 5231
5.8% 934
5.5% 972
0.1% 559
0.2% 545
pS6 Ser235/236
B
Glucose
No glucose
Control
GSK3 inh
IFN-γ
CD107a
0.26
0.11
0.20
0.11
% total HIV Gag-specific response in CD8+ T cells
*
P=0.06
Control
GSK3 inh
Glucose
No glucose
C
No glucose : glucose ratio % total HIV Gag-specific response
NS
Control
GSK3 inh
D
% TNF-α+ HIV Gag-specific cells in the absence of glucose
*
Control
GSK3 inh

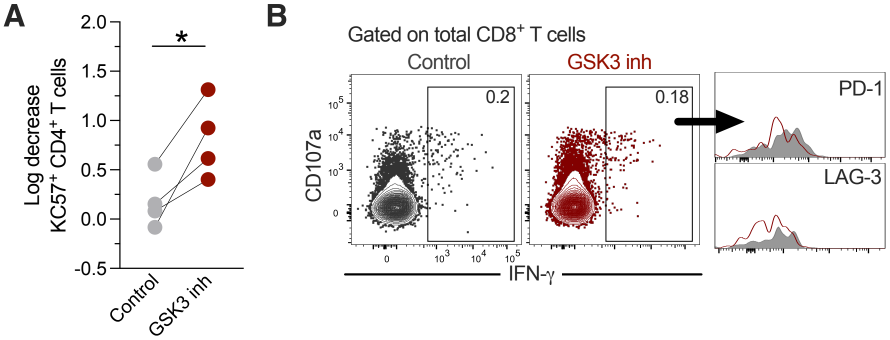

A
2.0
1.5
1.0
0.5
0.0
-0.5
*
Log decrease
KC57+ CD4+ T cells
Control
GSK3 inh
B
Gated on total CD8+ T cells
Control
GSK3 inh
0.2
0.18
CD107a
IFN-γ
PD-1
LAG-3

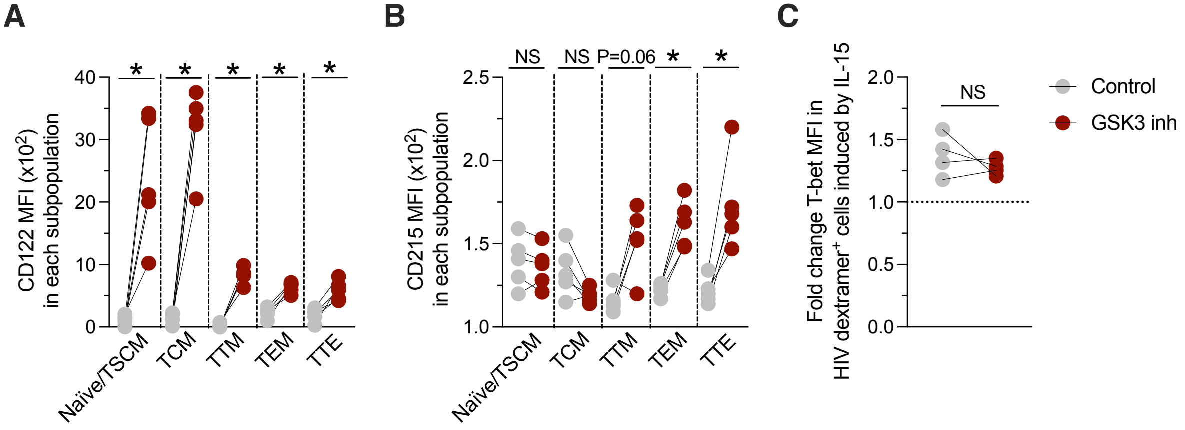
A
B
C
CD122 MFI (x10^2) in each subpopulation
CD215 MFI (x10^2) in each subpopulation
Fold change T-bet MFI in HIV dextramer+ cells induced by IL-15
*
NS
P=0.06
Naïve/TSCM
TCM
TTM
TEM
TTE
Control
GSK3 inh
